# Cusp of Non-Gaussian Density of Particles for a Diffusing Diffusivity Model

**DOI:** 10.3390/e23020231

**Published:** 2021-02-17

**Authors:** M. Hidalgo-Soria, E. Barkai, S. Burov

**Affiliations:** 1 Department of Physics, Institute of Nanotechnology and Advanced Materials, Bar-Ilan University, Ramat-Gan 5290002, Israel; Eli.Barkai@biu.ac.il; 2 Department of Physics, Bar-Ilan University, Ramat-Gan 5290002, Israel

**Keywords:** CTRW, diffusing-diffusivity, occupation time statistics

## Abstract

We study a two state “jumping diffusivity” model for a Brownian process alternating between two different diffusion constants, $D_{+}>D_{-}$, with random waiting times in both states whose distribution is rather general. In the limit of long measurement times, Gaussian behavior with an effective diffusion coefficient is recovered. We show that, for equilibrium initial conditions and when the limit of the diffusion coefficient $D_{-}⟶0$ is taken, the short time behavior leads to a cusp, namely a non-analytical behavior, in the distribution of the displacements P(x,t) for x⟶0. Visually this cusp, or tent-like shape, resembles similar behavior found in many experiments of diffusing particles in disordered environments, such as glassy systems and intracellular media. This general result depends only on the existence of finite mean values of the waiting times at the different states of the model. Gaussian statistics in the long time limit is achieved due to ergodicity and convergence of the distribution of the temporal occupation fraction in state $D_{+}$ to a δ-function. The short time behavior of the same quantity converges to a uniform distribution, which leads to the non-analyticity in P(x,t). We demonstrate how super-statistical framework is a zeroth order short time expansion of P(x,t), in the number of transitions, that does not yield the cusp like shape. The latter, considered as the key feature of experiments in the field, is found with the first correction in perturbation theory.

## 1. Introduction

The emergence of non-Gaussian features for the positional probability density function (PDF) of particle spreading, denoted P(x,t), in a disordered environment is a common attribute that arises in many different physical and biological systems. Specifically, a tent like shape of the PDF, in the semi-log scale, together with a linear time dependence of the mean square displacement (MSD) appear for diffusion in glassy system [1], biological cells [2,3,4,5,6,7], and colloidal suspensions [8,9,10,11]. This tent shape, sometimes fitted with a Laplace distribution P(x,t)∼exp(−C|x|) with *C* a constant, suggests that the decay of the PDF is exponential. This feature is becoming a more frequent observation for the spreading of molecules. Phenomenological approaches are diffusing diffusivity models, in which non-Gaussianity is obtained by coupled stochastic differential equations with random diffusion coefficients [7,12,13,14,15,16,17,18,19,20], and path integrals formalism for Brownian motion in the presence of a sink [21]. More recently, theoretical frameworks describing this behavior emerged from continuous time random walk (CTRW) approaches employing large deviations theory [22,23,24] and microscopical models like molecular dynamics of tracer particles in polymer networks [25,26] and interacting particles with fluctuating sizes [27,28,29], the so-called Hitchhiker model [28].

While in some of the systems the non-Gaussian behavior disappears when the measurement time is made long enough, the short time tent-like decay of the PDF seems to be a universal phenomenon [22]. It is then natural to ask if there is some sort of universality that can be deduced for the temporal limit of short times. Within the diffusing diffusivity models for the large *x* limit, exponentially decaying propagators have been observed by employing a dichotomous process for the diffusivity [13]. The latter model consists of a “fast” and a “slow” phases, each one with a diffusion coefficient $D_{+}$ and $D_{-}$, respectively [13,30]. Furthermore, the appearance of a cusp at small displacements also has been reported in different diffusive approaches like the Sinai model [31], employing the quenched trap model [32,33,34,35,36,37] or spatial dependence in the diffusivity [38], within the Lévy–Lorentz gas model [39] and using the fractional Fokker–Planck equation [40]. It is important to notice that the cusp found in [31,32,33,34,36,38,39,40] is within the context of anomalous diffusion in the MSD sense, and those presented in [35,36,37] are for normal diffusive systems.

It is worth mentioning that several systems in nature exhibit, or can be reduced to, a dichotomous process. Examples of two state systems include nuclear magnetic imaging to measure the diffusion of heterogeneous molecules [41], diffusion in glassy materials [1], blinking quantum dots [42,43], diffusion in single molecules tracking experiments [4,7], and protein conformational dynamics [44]. Other approaches for analyzing two state systems were also devised over the years; see heterogeneous molecular transport [41], telegraphic noise [43], Lèvy Flights [45], and CTRW models [1,22].

In this work, we deal with a two state jumping diffusivity model with equilibrium initial conditions, i.e., we assume that the process started long before the measurement began. The long measurement time behavior of the positional PDF for this model is Gaussian and is independent of the specifics of the waiting times at the different diffusive states. A rather unexpected result is achieved for the opposite temporal regime. We obtain that the behavior in the limit of the short measurement times and the shape of the positional PDF of the molecule spreading in the two state jumping diffusivity model attains a cusp or a general tent-like shape. Our result is based on the statistics of the temporal occupation fraction of the diffusivity states, the latter is defined as the time spent in state $D_{+}$ over the total measurement time. The Gaussian behavior in the long measurement time is dictated by the δ-function shape of the distribution of this temporal occupation fraction, a feature that is solely based on the ergodic properties of the system. We show that, in the limit of short measurement time, the distribution of the temporal occupation fraction attains a uniform distribution that leads to the mentioned cusp behavior of P(x,t). The uniformity of the occupation fraction is a general result in the sense that it does not depend on the statistics of the waiting times in the two states, and the latter can be arbitrary. The non-Gaussian behavior of P(x,t) for short measurement times is similarly general as the Gaussian behavior of the propagator for long times. We then show that our approach reproduces the results of a specific representative system with exponentially distributed waiting times.

Our manuscript is organized as follows: in Section 2.1, we introduce the jumping diffusivity model and the initial conditions utilized in this work. In Section 2.2, we develop our theory for the statistics of the occupation time in the short measurement time limit, for which the PDFs of the waiting times in states $D_{\pm }$ are rather general. The obtained behavior of the occupation fraction is used in order to describe the non-Gaussian features of P(x,t), i.e., its cusp shape, that is observed in this model. In Section 2.3, we corroborate our previous results for a system with exponentially distributed waiting times. In Section 3.1, we discuss briefly how these theoretical results differ from those found within the super-statistical approach [12,14,21] and further how our approach may be applicable in experiments. Finally, in Section 4, we present a summary of our results, and we discuss briefly recent work of Postnikov et al. [37] who considered a model with quenched disorder, emphasizing the importance of equilibrium initial conditions. The main derivations are given in the corresponding Appendixes.

## 2. Results

### 2.1. The Model

**Figure 1:**
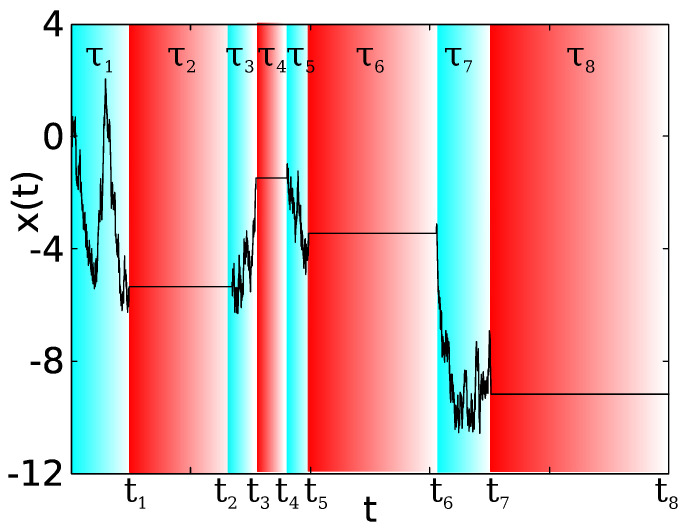
Typical trajectory of x(t) given by Equation (2) with $D_{+}=10$ (blue regions), and $D_{-}=0$ (red regions). For this trajectory, exponential waiting times with ${\langle \tau \rangle }_{+}=1$ and ${\langle \tau \rangle }_{-}=5$ were used.

**Figure 2:**
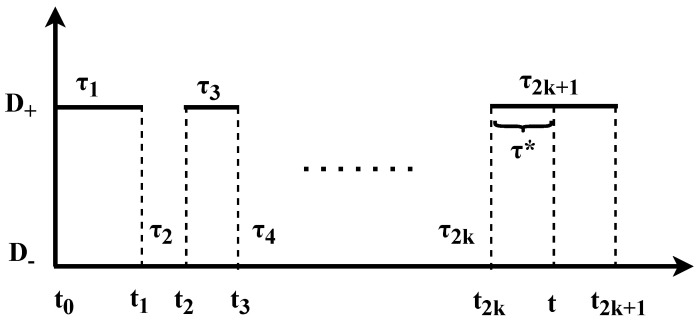
Alternating process for the diffusivity, starting from the state ‘+’ and N=2k+1. For the case of equilibrium initial conditions exposed in Section 2.2, for N=1, $\tau_{1}$ works as the forward recurrence time with PDF Equation (10).

We consider a two state renewal model, with a stochastic diffusion field D(t) for a particle in a random medium. The position of the particle is following a diffusion process given by $dx\left(t\right)/dt=\sqrt{2D(t)}\xi$. $D\left(t\right)\in \left\{D_{+},D_{-}\right\}$ is a dichotomous model, considering the case when $D_{-}<D_{+}$ and ξ is a standard white noise, i.e., with mean zero, variance one, and delta correlated. As an example of the dynamics of the model, at a given time, the particle follows a pure diffusion process with a diffusion coefficient $D_{+}>0$ during a period τ. After this time period has elapsed, the diffusion coefficient jumps and, during the next time interval, the particle diffuses with diffusion coefficient $D_{-}$. The waiting times at each state $D_{\pm }$ are distributed according to a general PDF $\psi_{\pm }\left(\tau \right)$, with mean waiting times ${\langle \tau \rangle }_{\pm }$. The subscript ± denotes whether the waiting times are defined for the $D_{+}$ or $D_{-}$ states. In the following, we present the two-state model with $D_{-}=0$, while the case with $D_{+}>D_{-}>0$ is analyzed in Appendix A. In Figure 1, we show representative trajectories for the position at time *t*, x(t), while, in Figure 2, we present the same for D(t) and we show the notation we use.

We define $T_{\pm }$ as the occupation time in state “±”, namely the total amount of time that the process diffuses with $D_{+}$ or $D_{-}$ during *t*. Jumps between states $D_{+}$ and $D_{-}$ occur at random times $t_{1}$, $t_{2}$, etc., until a final measurement time *t* and clearly $t=T_{+}+T_{-}$. The intervals of time between each jump are defined by $\tau_{1}=t_{1}$, $\tau_{2}=t_{2}-t_{1}$, $\tau_{3}=t_{3}-t_{2}$, etc., see Figure 2. Then, the occupation times in each state, when started from $D_{+}$, are explicitly provided by

(1) $$\begin{array}{ccc} T_{+} & = & \tau_{1}+\tau_{3}+\ldots +\tau_{N}\\ T_{-} & = & \tau_{2}+\tau_{4}+\ldots +\tau_{N-1}+\tau^{*}\;\;\;\;if\;\;\;\;N=2k+1,\\ T_{+} & = & \tau_{1}+\tau_{3}+\ldots +\tau_{N-1}+\tau^{*},\\ T_{-} & = & \tau_{2}+\tau_{4}+\ldots +\tau_{N}\;\;\;\;\;\;\;\;\;\;\;\;\;\;\;\;\;\;\;\;\;\;\;\;if\;\;\;\;N=2k, \end{array}$$

where *N* is the random number of transitions that were performed between the two states during the measurement time *t* and *k* is an integer. The measurement time *t* and *N* satisfy $t\geq t_{N}$, with $t_{N}=\tau_{1}+\tau_{2}+\ldots +\tau_{N}$, i.e., the exact time when the *N*th jump was performed. The backward recurrence time $\tau^{*}$ is defined by $\tau^{*}=t-t_{N}$ [46]. Each waiting time $\tau_{i}$ follows $\tau_{i}=t_{i}-t_{i-1}$ with i∈(1,N). For this particular initial condition, odd values of *i* in $\tau_{i}$ refer to waiting times at $D_{+}$ and even values of *i* to waiting times during which the diffusion coefficient is $D_{-}$ (see Figure 2). Expressions similar to Equation (1) are also obtained when the process starts from $D_{-}$, see Equation (A65).

Since the particle is diffusing with a constant diffusion constant $D_{+}$ for time $\tau_{1}$, when starting from $D_{+}$, the position $x(\tau_{1})$ is simply $x\left(\tau_{1}\right)=\sqrt{2D_{+}\tau_{1}}\xi_{1}$, where $\xi_{1}$ is a zero mean Gaussian random variable with $\langle \xi_{1}^{2}\rangle =1$. When at the state with diffusion constant $D_{-}=0$, the particle is not moving, therefore $x\left(t_{2}\right)-x\left(t_{1}\right)=0$ and $x\left(t_{3}\right)-x\left(t_{2}\right)=\sqrt{2D_{+}\tau_{3}}\xi_{3}$, where $\xi_{3}$ is a zero mean Gaussian random variable with $\langle \xi_{3}^{2}\rangle =1$ independent of $\xi_{1}$. Generally, $x\left(t_{i}\right)-x\left(t_{i-1}\right)=\sqrt{2D_{\pm }\tau_{i}}\xi_{i}$, where all $\xi_{i}$ are independent zero mean Gaussian random variables that satisfy $\langle \xi_{i}^{2}\rangle =1$. By using Equation (1) and exploiting the properties of summation of independent Gaussian variables, we obtain that the position at general time *t* is provided by

(2) $$\begin{array}{c} x\left(t\right)=\sqrt{2D_{+}T_{+}}\xi , \end{array}$$

when $D_{+}>0$ and $D_{-}=0$. Equation (2) holds irrespective of the state at t=0. We see that the particles’ position is a product of two independent random variables, the square root of the time staying at the state $D_{+}$ times a standard Gaussian random variable.

In the following, we consider a situation in which the process has started long before the measurement began, i.e., at t=0, the process was already running for a very long time. In this way, the measurement begins from an initial condition in which the system is in equilibrium, meaning that the probability to start from $D_{+}$ is ${\langle \tau \rangle }_{+}/\left[{\langle \tau \rangle }_{+}+{\langle \tau \rangle }_{-}\right]$ and accordingly the probability to start from $D_{-}$ is ${\langle \tau \rangle }_{-}/\left[{\langle \tau \rangle }_{+}+{\langle \tau \rangle }_{-}\right]$ (see [46,47]). For this set-up, the PDF of the occupation time $T_{+}$, $f_{t}\left(T_{+}\right)$, is determined by the contribution to start from $D_{+}$ and the contribution to start from $D_{-}$, yielding

(3) $$\begin{array}{c} f_{t}\left(T_{+}\right)=\frac{{\langle \tau \rangle }_{+}}{{\langle \tau \rangle }_{+}+{\langle \tau \rangle }_{-}}f_{t}^{+}\left(T_{+}\right)+\frac{{\langle \tau \rangle }_{-}}{{\langle \tau \rangle }_{+}+{\langle \tau \rangle }_{-}}f_{t}^{-}\left(T_{+}\right), \end{array}$$

where $f_{t}^{\pm }\left(T_{+}\right)$ is the PDF of $T_{+}$ for measurement time *t*, given that the process has started from ±. Since $D_{-}=0$, Equation (2) dictates that the positional PDF, provided that the system has occupied the state with $D_{+}$ for a time $T_{+}$, is given by

(4) $$\begin{array}{c} P\left(x|T_{+}\right)=\frac{e^{-\frac{x^{2}}{4D_{+}T_{+}}}}{\sqrt{4\pi D_{+}T_{+}}}. \end{array}$$

The propagator of the system is obtained via integrating over all possible values of the occupation time $T_{+}$, whose PDF is $f_{t}\left(T_{+}\right)$ Equation (3), yielding

(5) $$\begin{array}{ccc} P(x,t) & = & \int_{0}^{t}P\left(x|T_{+}\right)f_{t}\left(T_{+}\right)dT_{+}. \end{array}$$

Likewise, we can work with the temporal occupation fraction, which is defined by $p_{+}=T_{+}/t$ with $0\leq p_{+}\leq 1$. In this case, the positional PDF for a specific value of $p_{+}$ follows

(6) $$\begin{array}{c} P\left(x|p_{+}\right)=\frac{e^{-\frac{x^{2}}{4D_{+}tp_{+}}}}{\sqrt{4\pi D_{+}tp_{+}}}. \end{array}$$

and the propagator is obtained similarly to Equation (5), but using the PDF of $p_{+}$, which we denote by $g_{t}\left(p_{+}\right)$,

(7) $$\begin{array}{ccc} P(x,t) & = & \int_{0}^{1}P\left(x|p_{+}\right)g_{t}\left(p_{+}\right)dp_{+}. \end{array}$$

Since the properties of $P(x|T_{+})$ or $P(x|p_{+})$ are known, the task of computing the propagator completely depends on our ability to calculate the PDF of $T_{+}$ or $p_{+}$. In the following section, we address this problem.

### 2.2. The General Case: Arbitrary Distribution of Waiting Times

Two regimes of the process are of special interest. The long and the short limits of the measurement time *t*. The two different limits involve different considerations when computing the PDFs of the occupation time ($T_{+}$) and fraction ($p_{+})$. We first handle the regime of small *t* and then we treat the t→∞ limit.

#### 2.2.1. Short Time Regime

The PDF of the occupation time $T_{+}$ is defined by Equation (3). We condition on the number of transitions *N*, and each term $f_{t}^{\pm }\left(T_{+}\right)$ is provided by

(8) $$\begin{array}{c} f_{t}^{\pm }\left(T_{+}\right)=\sum_{N=0}^{\infty }f_{t}^{\pm }\left(T_{+}|N\right)Q_{t}^{\pm }\left(N\right), \end{array}$$

where $Q_{t}^{\pm }\left(N\right)$ is the probability to perform exactly *N* transitions during *t* when the process started at ±. $f_{t}^{\pm }\left(T_{+}|N\right)$ is the PDF of $T_{+}$ when exactly *N* transitions were performed (during *t*), and the process has started from ±. This conditional probability is obtained by counting the number of trajectories of temporal span *t* that started from the ± state and performed exactly *N* transitions, out of the total number of trajectories that started from the ± state and for which the diffusion spent a total time $T_{+}$ at this state. Utilizing Equation (8), we rewrite Equation (3) as

(9) $$\begin{array}{c} f_{t}\left(T_{+}\right)=\frac{{\langle \tau \rangle }_{+}}{{\langle \tau \rangle }_{+}+{\langle \tau \rangle }_{-}}\sum_{N=0}^{\infty }f_{t}^{+}\left(T_{+}|N\right)Q_{t}^{+}\left(N\right)+\frac{{\langle \tau \rangle }_{-}}{{\langle \tau \rangle }_{+}+{\langle \tau \rangle }_{-}}\sum_{N=0}^{\infty }f_{t}^{-}\left(T_{+}|N\right)Q_{t}^{-}\left(N\right). \end{array}$$

Since we consider a renewal process, the expression for $Q_{t}^{\pm }\left(N\right)$ is known in the Laplace space [42], as ${\hat{Q}}_{s}^{\pm }\left(N\right)=L\left\{Q_{t}^{\pm }\left(N\right)\right\}=\int_{0}^{\infty }Q_{t}^{\pm }\left(N\right)\exp\left(-ts\right)\;dt$, for any general ${\hat{\psi }}_{\pm }\left(s\right)=L^{}\left\{\psi_{\pm }\left(\tau \right)\right\}$. Concretely, $Q_{t}^{\pm }\left(N\right)$ is obtained by taking into account all the possibilities to perform *N* jumps up to time $t_{N}<t$, and no additional jumps during the backward recurrence time $\tau^{*}$. This sums up to a convolution of N+1 random variables. It is important to notice that, since we assume equilibrium initial conditions, $\tau_{1}$, which is measured from t=0, is only a part of a full renewal event and is termed the forward recurrence time. The PDF of $\tau_{1}$ for the ± state, $f_{eq}^{\pm }\left(\tau_{1}\right)$, is provided by (see [46])

(10) $$f_{eq}^{\pm }\left(\tau_{1}\right)=\left(1-\int_{0}^{\tau_{1}}\psi_{\pm }\left(\tau \right)\;d\tau \right)/{\langle \tau \rangle }_{\pm }$$

and in the Laplace space $L\left\{f_{eq}^{\pm }\left(\tau_{1}\right)\right\}=\left(1-{\hat{\psi }}_{\pm }\left(s\right)\right)/{\langle \tau \rangle }_{\pm }s$. This initial condition stems from the equilibrium of the underlying process, in which we do not have a jump at the initial time ($t_{0}=0$ in Figure 2). In the literature [13,42,46,48,49], the case where the renewal process starts at t=0 is called ordinary or non-equilibrium, and as we will see below, by following our approach, this does not yield any universal features for P(x,t), hence the assumption of an equilibrium process is important in our methodology, (see discussion about non-equilibrium initial conditions in Appendix B).

The probability of not performing any jumps during $\tau^{*}$ is equivalent to the probability of obtaining a waiting time $\tau_{N+1}>\tau^{*}$, i.e., $1-\int_{0}^{\tau^{*}}\psi_{\pm }\left(\tau \right)\;d\tau$. Eventually, by implementing the initial equilibrium condition, we obtain

(11) $$\begin{array}{ccc} {\hat{Q}}_{s}^{\pm }\left(0\right) & = & \frac{1-\frac{1-{\hat{\psi }}_{\pm }\left(s\right)}{{\langle \tau \rangle }_{\pm }s}}{s},\\ {\hat{Q}}_{s}^{\pm }\left(1\right) & = & \left(\frac{1-{\hat{\psi }}_{\pm }\left(s\right)}{{\langle \tau \rangle }_{\pm }s}\right)\left(\frac{1-{\hat{\psi }}_{\mp }\left(s\right)}{s}\right),\\ {\hat{Q}}_{s}^{\pm }\left(2\right) & = & \left(\frac{1-{\hat{\psi }}_{\pm }\left(s\right)}{{\langle \tau \rangle }_{\pm }s}\right){\hat{\psi }}_{\mp }\left(s\right)\left(\frac{1-{\hat{\psi }}_{\pm }\left(s\right)}{s}\right),\\ {\hat{Q}}_{s}^{\pm }\left(3\right) & = & \left(\frac{1-{\hat{\psi }}_{\pm }\left(s\right)}{{\langle \tau \rangle }_{\pm }s}\right){\hat{\psi }}_{\mp }\left(s\right)\psi_{\pm }\left(s\right)\left(\frac{1-{\hat{\psi }}_{\mp }\left(s\right)}{s}\right). \end{array}$$

In all the equations above on the right-hand side, we have a multiplication of functions in the Laplace space, this implies convolutions as we transform from *s* to *t*. The first term in the multiplication on the right-hand side of Equation (11) obviously stems from the equilibrium initial condition under study. We assume that the PDF of the waiting times is analytic for τ→0, thus we can express $\psi_{\pm }\left(\tau \right)$ as [22,23]

(12) $$\begin{array}{c} \psi_{\pm }\left(\tau \right)∼C_{A_{\pm }}^{\pm }\tau^{A_{\pm }}+C_{A_{\pm }+1}^{\pm }\tau^{A_{\pm }+1}+\ldots , \end{array}$$

with $A_{\pm }\geq 0$ an integer number. As an example, consider the case with exponential waiting times, i.e., $\psi_{\pm }\left(\tau \right)=\psi \left(\tau \right)=\exp\left(-\tau /\langle \tau \rangle \right)/\langle \tau \rangle$, namely the waiting times at the $D_{\pm }$ states are identically distributed. Its analytic expansion is $\psi \left(\tau \right)∼1/\langle \tau \rangle -\tau /{\langle \tau \rangle }^{2}$, with $A_{\pm }=0$, $C_{A_{\pm }}^{\pm }=1/\langle \tau \rangle$ and $C_{A_{\pm }+1}^{\pm }=1/{\langle \tau \rangle }^{2}$. The analyticity of $\psi_{\pm }\left(\tau \right)$ Equation (12) is a very mild demand that covers a wide range of sojourn times distributions. Since we are interested in the small *t* limit, the corresponding behavior in the Laplace space is found for s→∞, where the leading terms of ${\hat{\psi }}_{\pm }\left(s\right)$ are [22]

(13) $$\begin{array}{c} {\hat{\psi }}_{\pm }\left(s\right)∼\frac{\Gamma \left(A_{\pm }+1\right)C_{A_{\pm }}^{\pm }}{s^{A_{\pm }+1}}+\frac{\Gamma \left(A_{\pm }+2\right)C_{A_{\pm }+1}^{\pm }}{s^{A_{\pm }+2}}+\ldots , \end{array}$$

For the mentioned example with exponential waiting times, $\hat{\psi }\left(s\right)∼1/\left[\langle \tau \rangle s\right]$. Using Equation (13) for ${\hat{Q}}_{s}^{\pm }\left(N\right)$, we obtain that, in the s→∞ limit, corresponding to the short time limit, which is at the focus of our interest

(14) $$\begin{array}{ccc} {\hat{Q}}_{s}^{\pm }\left(0\right) & ∼ & \frac{1}{s}-\frac{1}{{\langle \tau \rangle }_{\pm }s^{2}}+\frac{\Gamma \left(A_{\pm }+1\right)C_{\pm }^{\pm }}{{\langle \tau \rangle }_{\pm }s^{A_{\pm }+3}}+\ldots ,\\ {\hat{Q}}_{s}^{\pm }\left(1\right) & ∼ & \frac{1}{{\langle \tau \rangle }_{\pm }s^{2}}-\frac{2C_{A_{\pm }}^{\pm }\Gamma \left(A_{\pm }+1\right)}{{\langle \tau \rangle }_{\pm }s^{A_{\pm }+3}}+\ldots \\ {\hat{Q}}_{s}^{\pm }\left(2\right) & ∼ & \frac{\Gamma \left(A_{\mp }+1\right)C_{A_{\mp }}^{\mp }}{{\langle \tau \rangle }_{\pm }s^{A_{\mp }+3}}+\ldots \\ {\hat{Q}}_{s}^{\pm }\left(3\right) & ∼ & \frac{\Gamma \left(A_{\pm }+1\right)\Gamma \left(A_{\mp }+1\right)C_{A_{\pm }}^{\pm }C_{A_{\mp }}^{\mp }}{{\langle \tau \rangle }_{\pm }s^{A_{\pm }+A_{\mp }+4}}+\ldots . \end{array}$$

We see that the leading terms for all ${\hat{Q}}_{s}^{\pm }\left(N\right)$ with N>1 are of the order $1/s^{\gamma }$ with γ>2. Thus, in the small *t* limit, terms with N>1 contain contributions that scale like $t^{\gamma -1}$ and are negligible with respect to the N∈{0,1} cases. Therefore, only the first two $Q_{t}^{\pm }\left(N\right)$s are taken into account, i.e.,

(15) $$\begin{array}{ccc} Q_{t}^{\pm }\left(0\right) & ∼ & 1-\frac{t}{{\langle \tau \rangle }_{\pm }}, \end{array}$$

(16) $$\begin{array}{ccc} Q_{t}^{\pm }\left(1\right) & ∼ & \frac{t}{{\langle \tau \rangle }_{\pm }}. \end{array}$$

This is an expected result, as, for short times, only contributions from a single transition and zero transitions are important. By calculating $Q_{t}^{\pm }\left(N\right)$, we advanced towards obtaining the behavior of the PDF of $T_{+}$, according to Equation (9), in order to complete this mission, one needs to compute the relevant contributions of $f_{t}^{\pm }\left(T_{+}|N\right)$ in the t→0 limit. First, we see that the conditional distribution $f_{t}^{\pm }\left(T_{+}|0\right)$ depends only on the starting state. There are only two types of trajectories that have performed 0 transitions, i.e., for all the time, they have been either at $D_{+}$ or at $D_{-}$. Consequently,

(17) $$\begin{array}{ccc} f_{t}^{+}\left(T_{+}|0\right) & = & \delta (t-T_{+}), \end{array}$$

(18) $$\begin{array}{ccc} f_{t}^{-}\left(T_{+}|0\right) & = & \delta (T_{+}). \end{array}$$

The calculation of $f_{t}^{\pm }\left(T_{+}|N\right)$ is obtained by conditioning over the first event. If starting from the + state, the process will spend a time $\tau_{1}$ at this state before jumping to the − state. $\tau_{1}$ can attain any value $0\leq \tau_{1}\leq T_{+}$ and for the remaining time $t-\tau_{1}$ the process has to perform one transition less. In general, without regarding the initial conditions of the problem, an integration over all possible $\tau_{1}$’s provides the relation

(19) $$f_{t}^{+}\left(T_{+}|N^{\prime }+1\right)=\int_{0}^{T_{+}}\frac{1}{B_{+}}\psi_{+}\left(\tau_{1}\right)f_{t-\tau_{1}}^{-}\left(T_{+}-\tau_{1}|N^{\prime }\right)\;d\tau_{1}$$

with $N^{\prime }+1=N$, and $B_{+}$ a normalization factor. For instance, for N=1, we have that $\int_{0}^{t}\psi_{+}\left(\tau_{1}\right)/B_{+}\;d\tau_{1}=1$, which stems from the fact that we consider only trajectories of time span *t*. The corresponding formula for $f_{t}^{-}\left(T_{+}|N^{\prime }+1\right)$ is

(20) $$f_{t}^{-}\left(T_{+}|N^{\prime }+1\right)=\int_{0}^{t-T_{+}}\frac{1}{B_{-}}\psi_{-}\left(\tau_{1}\right)f_{t-\tau_{1}}^{+}\left(T_{+}|N^{\prime }\right)\;d\tau_{1}.$$

Since we are assuming equilibrium initial conditions, the $\psi_{\pm }$ in the $N^{\prime }+1$ element of the iterative forms (Equations (19) and (20)) must be replaced by $f_{eq}^{\pm }$ (Equation (10)). As was already noted above, only the N=0 and N=1 are of interest in the small *t* limit, then, according to Equations (17), (20), and (10), we get for N=1

(21) $$\begin{array}{ccc} f_{t}^{+}\left(T_{+}|1\right) & = & \frac{f_{eq}^{+}\left(T_{+}\right)}{\int_{0}^{t}f_{eq}^{+}\left(t^{\prime }\right)\;dt^{\prime }}, \end{array}$$

(22) $$\begin{array}{ccc} f_{t}^{-}\left(T_{+}|1\right) & = & \frac{f_{eq}^{-}\left(t-T_{+}\right)}{\int_{0}^{t}f_{eq}^{-}\left(t^{\prime }\right)\;dt^{\prime }}. \end{array}$$

Using the small time approximation of $\psi_{\pm }\left(\tau \right)$ Equation (12), in Equations (21) and (22), we obtain that, independently of the starting state,

(23) $$\begin{array}{c} f_{t}^{\pm }\left(T_{+}|1\right)∼\frac{1}{t}. \end{array}$$

The 1/t dependence comes from the integral factors in Equations (21) and (22), all the other terms in the numerator and denominator simply cancel out. See Appendix B for a complementary derivation of Equation (23) using the definition of the joint PDF of $T_{+}$ and *N*. Gathering Equations (15)–(18), and (23) in Equation (9), we find that

(24) $$\begin{array}{ccc} f_{t}\left(T_{+}\right) & ∼ & \frac{{\langle \tau \rangle }_{+}}{{\langle \tau \rangle }_{+}+{\langle \tau \rangle }_{-}}\left(1-\frac{t}{{\langle \tau \rangle }_{+}}\right)\delta \left(t-T_{+}\right)\\ & + & \frac{{\langle \tau \rangle }_{-}}{{\langle \tau \rangle }_{+}+{\langle \tau \rangle }_{-}}\left(1-\frac{t}{{\langle \tau \rangle }_{-}}\right)\delta \left(T_{+}\right)+\frac{2}{\langle \tau_{+}\rangle +\langle \tau_{-}\rangle }. \end{array}$$

The PDF of the occupation fraction is obtained trivially from Equation (24) by changing variables to $p_{+}=T_{+}/t$

(25) $$\begin{array}{ccc} g_{t}\left(p_{+}\right) & ∼ & \frac{{\langle \tau \rangle }_{+}}{{\langle \tau \rangle }_{+}+{\langle \tau \rangle }_{-}}\left(1-\frac{t}{{\langle \tau \rangle }_{+}}\right)\delta \left(1-p_{+}\right)\\ & + & \frac{{\langle \tau \rangle }_{-}}{{\langle \tau \rangle }_{+}+{\langle \tau \rangle }_{-}}\left(1-\frac{t}{{\langle \tau \rangle }_{-}}\right)\delta \left(p_{+}\right)+\frac{2t}{{\langle \tau \rangle }_{+}+{\langle \tau \rangle }_{-}}. \end{array}$$

The third term in Equations (24) and (25) is uniform, i.e., terms which are independent of $T_{+}$ or $p_{+}$, and this is the first main result of this paper. All the additional terms and contributions to the PDF of $p_{+}$ only introduce terms that depend on higher orders of *t* and are thus negligible in the small *t* limit. This means that, for equilibrium initial conditions, regardless of the exact form of $\psi_{\pm }\left(\tau \right)$, the PDF of $p_{+}$ (Equation (25)) is always uniform for $0<p_{+}<1$. This general uniform behavior of the PDF of the occupation fraction is applicable for an extremely large class of waiting times PDFs $\psi_{\pm }\left(\tau \right)$. As a remark, the connection between the conditional PDF of $T^{+}$, $f_{t}^{\pm }\left(T_{+}|N\right)$, and the joint PDF of $T^{+}$ and *N*, $f_{t}^{\pm }\left(T_{+},N\right)$ is discussed in Appendix B.1. In the following, it is shown that this uniformity leads to universal features of the propagator in the limit of small *t*. In Section 2.3.1 and Section 2.3.2, we treat particular examples (with exponential waiting times) that are exactly tractable, without any simplifications or assumptions. The results agree perfectly with the general form in Equation (25). It is important to notice that our approximations affect only the form of $g_{t}\left(p_{+}\right)$ and do not affect $P(x|p_{+})$. This allows us to obtain the behavior of P(x,t) for any −∞<x<∞, as is shown below.

##### 2.2.1.1. P(x,t) for Arbitrary Waiting Times

In order to obtain the positional PDF for small *t*, we combine Equations (6), (7), and (25), which, after integration, gives

(26) $$\begin{array}{ccc} P(x,t) & = & \frac{{\langle \tau \rangle }_{+}}{{\langle \tau \rangle }_{+}+{\langle \tau \rangle }_{-}}\left(1-\frac{t}{{\langle \tau \rangle }_{+}}\right)\frac{e^{-\frac{x^{2}}{4D_{+}t}}}{\sqrt{4\pi D_{+}t}}+\frac{{\langle \tau \rangle }_{-}}{{\langle \tau \rangle }_{+}+{\langle \tau \rangle }_{-}}\left(1-\frac{t}{{\langle \tau \rangle }_{-}}\right)\delta \left(x\right)\\ & + & \left(\frac{2t}{{\langle \tau \rangle }_{+}+{\langle \tau \rangle }_{-}}\right)\left\{\frac{e^{-\frac{x^{2}}{4D_{+}t}}}{\sqrt{\pi D_{+}t}}-\frac{\left|x\right|}{2D_{+}t}\left(1-Erf\left(\frac{\left|x\right|}{\sqrt{4D_{+}t}}\right)\right)\right\}. \end{array}$$

**Figure 3:**
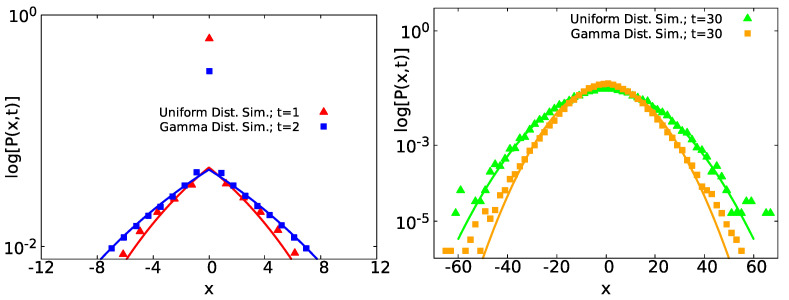
Distribution of displacements P(x,t) in semi-log scale, obtained by simulations, for a two state system with uniform distributed waiting times and gamma distributed waiting times. The left panel presents short time results where a tent like shape is clearly visible and a non-analytical feature is obvious, while the right panel exhibits Gaussian statistics for long times. Left: P(x,t) for t=1 for τ∼U(0,5) at $D_{+}$ and τ∼U(0,10) at $D_{-}$ (red triangles)—with ${\langle \tau \rangle }_{+}=2.5<{\langle \tau \rangle }_{-}=5$. In addition, t=2 with τ∼Gamma(0.5,8) at $D_{+}$ and τ∼Gamma(0.5,12) at $D_{-}$ (blue squares), such that ${\langle \tau \rangle }_{+}=4<{\langle \tau \rangle }_{-}=6$. Both cases fit with Equation (26) (red and blue solid lines) with a tent like shape. In both normalized histograms at x=0, there is a peak representing the Dirac delta function in Equation (26). Right: P(x,t) for t=30 and waiting times uniformly distributed (green triangles) with the same parameters as above and for gamma distributed waiting times (orange squares) with τ∼Gamma(2,1) at $D_{+}$, τ∼Gamma(8,1) at $D_{-}$, and ${\langle \tau \rangle }_{+}=2<{\langle \tau \rangle }_{-}=8$. We employed the last set of parameters in the gamma distributed waiting times in order to avoid an overlapping between curves. P(x,t) converges to the Gaussian statistics Equation (29) (green and orange solid lines). In all the presented cases, $D_{+}=10$ and $D_{-}=0$ were used.

Considering x≠0, in the limit of x⟶0 when $\exp\left(-x^{2}/4D_{+}t\right)∼1-x^{2}/4D_{+}t$ and $1-Erf(|x|/\sqrt{4D_{+}t})∼1-2|x|/\sqrt{4\pi D_{+}t}$. After substituting in Equation (26), it turns into

(27) $$\begin{array}{ccc} P(x,t) & ∼ & \frac{\left(3t+{\langle \tau \rangle }_{+}\right)}{\sqrt{4\pi D_{+}t}\left[{\langle \tau \rangle }_{+}+{\langle \tau \rangle }_{-}\right]}-\frac{\left|x\right|}{D_{+}\left[{\langle \tau \rangle }_{+}+{\langle \tau \rangle }_{-}\right]}+K_{1}x^{2}, \end{array}$$

with $K_{1}=\left(5t-{\langle \tau \rangle }_{+}\right)/\left[8\left({\langle \tau \rangle }_{+}+{\langle \tau \rangle }_{-}\right)\sqrt{\pi }{\left(D_{+}t\right)}^{\frac{3}{2}}\right]$. We can see that, in Equation (27), there is a linear dependence on |x| in the vicinity of x=0. This means that for short enough measurement times the PDF of *x* will always have a tent like shape, irrespective of the distributions $\psi_{\pm }$ that were chosen (see Figure 3 below). Only the mean sojourn times affect the shape. This is a general result for the short time regime, and it is based on the general fact that the PDF of the temporal occupation fraction is uniform for $0<p_{+}<1$. Concretely, at short times when |x| is small, the decay of P(x,t) will always resemble an exponential one. For large |x|, the form of P(x,t) must be Gaussian, due to the fact that this limit is determined by the instances when no transition to $D_{-}$ was ever made and the transport is controlled by diffusion with $D_{+}$. However, if we only look at the particles that have moved, i.e., we get rid of the delta function at x=0 in Equation (26). We can relate these dynamics with some experiments which condition the measurements on the movement of the particles. This procedure is called population splitting; see [50,51]. Technically, if $D_{-}>0$, the cusp is not found; however, as long as $D_{-}/D_{+}<<1$, the tent like shape will be found; for further details, see Appendix A.

#### 2.2.2. Long Time Regime

In the limit t⟶∞, the PDF of the temporal occupation fraction $g_{t}\left(p_{+}\right)$ follows a different but also a general form. As mentioned, we are focusing on the case where both $\psi_{\pm }$ have finite first moments, ${\langle \tau \rangle }_{\pm }>0$. In the long time limit, ergodicity is satisfied, namely the equivalence of *ensemble* and temporal averages are attained. Particularly, in this case, the *ensemble* average of the occupation fraction at $D_{+}$ is equal to the temporal average which is defined by the fraction of average waiting times at $D_{+}$ and $D_{-}$, i.e., $\langle p_{+}\rangle ={\langle \tau \rangle }_{+}/\left[{\langle \tau \rangle }_{+}+{\langle \tau \rangle }_{-}\right]$ (see Appendix F). Thus, in the long time limit, $g_{t}\left(p_{+}\right)$ converges to a δ-function,

(28) $$\begin{array}{c} g_{t}\left(p_{+}\right)\overset{}{\underset{t\to \infty }{\to }}\delta \left(p_{+}-\frac{{\langle \tau \rangle }_{+}}{{\langle \tau \rangle }_{+}+{\langle \tau \rangle }_{-}}\right). \end{array}$$

Since ergodicity prevails, by using Equation (28) in Equation (7), the positional PDF gets the form

(29) $$\begin{array}{c} P\left(x,t\right)=\sqrt{\frac{{\langle \tau \rangle }_{+}+{\langle \tau \rangle }_{-}}{4\pi D_{+}t{\langle \tau \rangle }_{+}}}e^{-\frac{x^{2}\left({\langle \tau \rangle }_{+}+{\langle \tau \rangle }_{-}\right)}{4D_{+}t{\langle \tau \rangle }_{+}}}. \end{array}$$

In the long time limit, the positional PDF given by Equation (29) represents a Gaussian propagator with an effective diffusion coefficient $D_{+}{\langle \tau \rangle }_{+}/\left[{\langle \tau \rangle }_{+}+{\langle \tau \rangle }_{-}\right]$. Since ${\langle \tau \rangle }_{+}/\left[{\langle \tau \rangle }_{+}+{\langle \tau \rangle }_{-}\right]<1$, and the effective diffusion coefficient is always smaller compared with $D_{+}$. Indeed, this slow-down is an expected result due to the portion of the time that the particle spends in the state with $D_{-}=0$ and basically not moving during this period.

#### 2.2.3. Simulations

The two general limits of $g_{t}\left(p_{+}\right)$ Equations (25) and (28) produce two different prevailing distributions of P(x,t) Equations (26) and (29). In Figure 3, we compare analytical formulas Equations (26) and (29) (solid lines) with simulations of two different state models—one with uniform distributed waiting times τ∼U(0,5) for $D_{+}$ and τ∼U(0,10) for $D_{-}$ and with t=1 (red triangles) and t=30 (green triangles), such that ${\langle \tau \rangle }_{+}=2.5<{\langle \tau \rangle }_{-}=5$. Here, the notation τ∼U(a,b) means that τ has a uniform distribution with *a* and *b* the minimum and maximum values, respectively. In addition, the other with gamma distributed waiting times, such that τ∼Gamma(k,θ). The latter notation denotes that τ has a gamma distribution with *k* its shape parameter and θ the corresponding scale parameter. In this case, the PDF follows

(30) $$\begin{array}{c} \psi_{\pm }\left(\tau \right)=\frac{\tau^{k-1}e^{-\frac{\tau }{\theta }}}{\Gamma \left(k\right)\theta^{k}}, \end{array}$$

particularly the PDF of the gamma distribution Equation (30) implies a cumulative distribution function F(τ)=γ(k,τ/θ)/Γ(k), with γ(x,y) the incomplete gamma function and Γ(x) the standard gamma function. For the latter case, we used τ∼Gamma(0.5,8) at $D_{+}$ and τ∼Gamma(0.5,12) at $D_{-}$, for t=2 (blue squares) and t=30 (orange squares), with ${\langle \tau \rangle }_{+}=4<{\langle \tau \rangle }_{-}=6$. As we can see in the short time regime, for uniform and gamma distributed waiting times (red triangles and blue squares), P(x,t) has a tent shape for short displacements, and it agrees with Equation (26), joined with a peak at x=0 due to the Dirac delta function in Equation (26). For t=30 (green triangles and orange squares), each case of P(x,t) converges to Gaussian statistics (Equation (29)).

**Figure A2:**
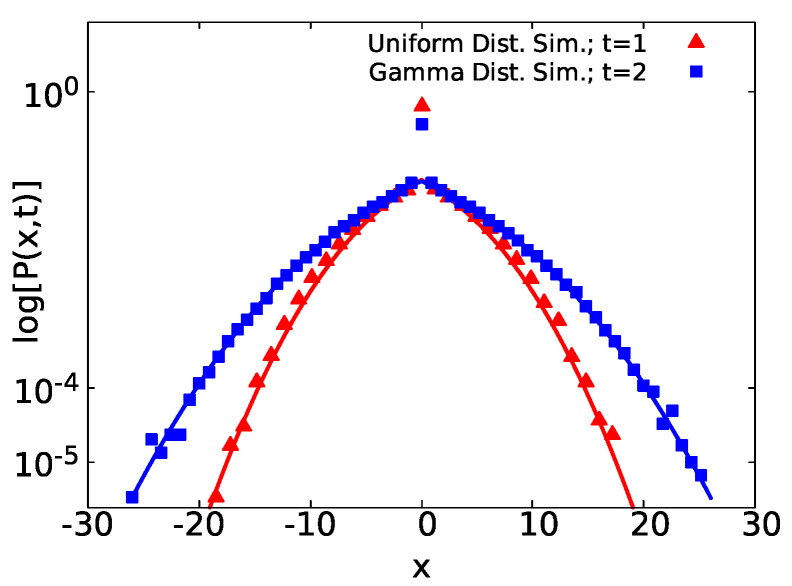
Distribution of displacements P(x,t) in semi-log scale, obtained from simulations, of a two state system with uniform and gamma distributed waiting times within the short time limit and displaying the whole span of *x*. P(x,t) for uniformly distributed waiting times is shown in red triangles. In addition, the case of gamma distributed waiting times is shown in blue squares. We employed the same set of parameters as those used in Figure 3 in the left panel. Both cases fit with Equation (26) (red and blue solid lines).

The cusp we have found for small |x| implies that we may approximate the distribution on a small scale with a Laplace like distribution, P(x,t)∼exp(−C|x|). However, clearly this does not hold globally for large *x*, see Figure A2 in Appendix C. Still within the interval of short displacements, due to the presence of the delta peak at the origin, we expect for this span a considerable contribution on the normalization of P(x,t). Particularly, we find that the area underneath the curve for the case of uniformly distributed waiting times (red line) in Figure 3 in the left panel has a value of 0.88 for x∈(−4,4). In addition, the corresponding area within the same figure, but, for gamma distributed waiting times, the (blue curve) has a value of 0.89 for x∈(−8,8).

### 2.3. Exponentially Distributed Waiting Times

In this section, we obtain $g_{t}\left(p_{+}\right)$ for a specific distribution of waiting times, but using different methods, which let us corroborate the validity of our general approach described above. We analyze the case of exponential waiting times in states with $D_{+}$ and $D_{-}$, each waiting time following a PDF given by

(31) $$\begin{array}{ccc} \psi_{\pm }\left(\tau \right) & = & \frac{e^{-\frac{\tau }{{\langle \tau \rangle }_{\pm }}}}{{\langle \tau \rangle }_{\pm }}. \end{array}$$

We show first the case of a two state system with the same mean waiting times and then investigate the complimentary case. In Appendix D, we analyze both cases for non-equilibrium initial conditions, e.g., a system starting from $D_{+}$.

#### 2.3.1. Equal Mean Waiting Times ${\langle \tau \rangle }_{+}={\langle \tau \rangle }_{-}$

Let us consider a system with ${\langle \tau \rangle }_{+}={\langle \tau \rangle }_{-}=\langle \tau \rangle$. We know that the temporal fraction occupation $p_{+}$ and $T_{+}$ can be related to the difference of occupation times defined by $S_{t}=T_{+}-T_{-}$, as $S_{t}=2T_{+}-t=2p_{+}t-t$ [46]. In this section, we analyze the double Laplace transform of the PDF of $S_{t}$, called $\phi_{t}\left(S_{t}\right)$, with Laplace pairs $S_{t}\Leftrightarrow v$ and t⇔s. In [46], $\phi_{t}\left(S_{t}\right)$ is provided by

(32) $$\begin{array}{c} {\hat{\phi }}_{s}\left(v\right)=\frac{s[1-\psi (s+v)\psi (s-v)]+v[\psi (s+v)-\psi (s-v)]}{\left(s^{2}-v^{2}\right)\left[1-\psi \left(s+v\right)\psi \left(s-v\right)\right]}. \end{array}$$

The Laplace transform of ψ(τ) in Equation (31) is given by $L\left\{\psi (\tau )\right\}=\hat{\psi }\left(s\right)=\frac{1}{1+\langle \tau \rangle s}$. Substituting $\hat{\psi }\left(s\right)$ in Equation (32), we obtain

(33) $$\begin{array}{c} {\hat{\phi }}_{s}\left(v\right)=\frac{s+2\langle \tau \rangle }{s^{2}+2\langle \tau \rangle s-v^{2}}. \end{array}$$

In Appendix E, an analytical expression for the PDF of $S_{t}$ is found, i.e., inverse Laplace transform of Equation (33) is performed (see Equation (A44)). Then, remember that the temporal occupation fraction in the plus state $p_{+}$ is related to the difference of occupation times as $S_{t}=2p_{+}t-t$. We can employ Equation (A44) for obtaining the PDF of $p_{+}$, which is given by

(34) $$\begin{array}{ccc} g_{t}\left(p_{+}\right) & = & \frac{1}{2}e^{-\frac{t}{\langle \tau \rangle }}\{\delta \left(1-p_{+}\right)+\delta \left(p_{+}\right)\}\\ \; & + & \frac{t\Theta (t-|2p_{+}t-t\left|\right)e^{-\frac{t}{\langle \tau \rangle }}}{\langle \tau \rangle }\left[I_{0}\left(\frac{2t\sqrt{p_{+}\left(1-p_{+}\right)}}{\langle \tau \rangle }\right)+\frac{I_{1}\left(\frac{2t\sqrt{p_{+}\left(1-p_{+}\right)}}{\langle \tau \rangle }\right)}{2\sqrt{p_{+}\left(1-p_{+}\right)}}\right]. \end{array}$$

A similar expression for a system with non-equilibrium initial conditions (always starting from $D_{+}$) is found in Appendix D. Expanding Equation (34) in the short time limit t⟶0, i.e., t<<〈τ〉, Equation (34) can be approximated by a uniform distribution

(35) $$\begin{array}{c} g_{t}\left(p_{+}\right)∼\frac{e^{-\frac{t}{\langle \tau \rangle }}}{2}\{\delta \left(1-p_{+}\right)+\delta \left(p_{+}\right)\}+\frac{t}{\langle \tau \rangle }. \end{array}$$

**Figure 4:**
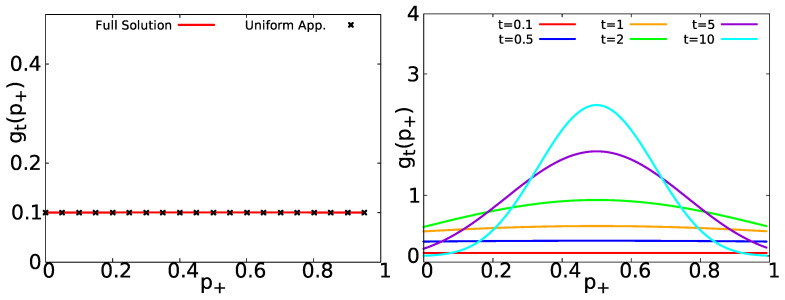
Left: Comparison between $g_{t}\left(p_{+}\right)$ Equation (34) (red solid line) and the short time uniform approximation Equation (35) (black asterisks) for exponentially distributed waiting times Equation (31) with ${\langle \tau \rangle }_{\pm }=\langle \tau \rangle =1$ and t=0.1. Right: $g_{t}\left(p_{+}\right)$ Equation (34) for 〈τ〉=1 and t∈{0.1,0.5,1,2,5,10}.

For ${\langle \tau \rangle }_{+}={\langle \tau \rangle }_{-}=\langle \tau \rangle$, Equation (35) agrees with Equation (25) obtained by the general approach of Section 2.2. In the left panel of Figure 4, we show the short time approximation of $g_{t}\left(p_{+}\right)$ (Equation (35)) compared with the general formula in Equation (34); it is evident that both results agree perfectly. In the right panel of Figure 4, we show Equation (34) for short and long measurement times. $g_{t}\left(p_{+}\right)$ evolves from a uniform distribution to a peaked distribution centered at its mean value $p_{+}=1/2$ (see Appendix E for a deduction of the central moments of $g_{t}\left(p_{+}\right)$).

##### Positional Distribution Function

An analytical expression for the positional distribution function P(x,t) (given by Equation (7)), with $g_{t}\left(p_{+}\right)$ provided by Equation (34), can be deduced by using the series representation of the modified Bessel functions, $I_{\nu }\left(y\right)=\sum_{k=0}^{\infty }{\left(\frac{y}{2}\right)}^{2k+\nu }/\left[k!\Gamma \left(\nu +k+1\right)\right]$. The integration in Equation (7) yields

(36) $$\begin{array}{cc} \; & P\left(x,t\right)=\frac{e^{-\frac{t}{\langle \tau \rangle }-\frac{x^{2}}{4Dt}}}{2\sqrt{4\pi Dt}}+\frac{\delta \left(x\right)e^{-\frac{t}{\langle \tau \rangle }}}{2}+\\ \; & \frac{te^{-\frac{t}{\langle \tau \rangle }-\frac{x^{2}}{4Dt}}}{2\langle \tau \rangle \sqrt{4\pi Dt}}\{\sum_{k=0}^{\infty }\frac{{\left(-1\right)}^{k}\pi }{k!}{\left(\frac{t}{\langle \tau \rangle }\right)}^{2k}\left[\frac{{}_{1}F_{1}\left(k+1;\frac{1}{2}-k;\frac{x^{2}}{4DT}\right)}{\Gamma \left(2k+\frac{3}{2}\right)\Gamma \left(\frac{1}{2}-k\right)}-\frac{{\left(\frac{x^{2}}{4Dt}\right)}^{k+\frac{1}{2}}\;{\;}_{1}F_{1}\left(2k+\frac{3}{2};k+\frac{3}{2};\frac{x^{2}}{4Dt}\right)}{\Gamma \left(k+1\right)\Gamma \left(k+\frac{3}{2}\right)}\right]+\\ \; & \frac{1}{2}\sum_{k=0}^{\infty }\frac{{\left(-1\right)}^{k}\pi }{(k+1)!}{\left(\frac{t}{\langle \tau \rangle }\right)}^{2k+1}\left[\frac{{}_{1}F_{1}\left(k+1;\frac{1}{2}-k;\frac{x^{2}}{4Dt}\right)}{\Gamma \left(2k+\frac{3}{2}\right)\Gamma \left(\frac{1}{2}-k\right)}-\frac{{\left(\frac{x^{2}}{4Dt}\right)}^{k+\frac{1}{2}}\;{\;}_{1}F_{1}\left(2k+\frac{3}{2};k+\frac{3}{2};\frac{x^{2}}{4Dt}\right)}{\Gamma \left(k+1\right)\Gamma \left(k+\frac{3}{2}\right)}\right]\}, \end{array}$$

with ${}_{1}F_{1}\left(a;b;z\right)$ the confluent hypergeometric function of the first kind. Nonetheless, in a short time limit, we can use the uniform approximation of $g_{t}\left(p_{+}\right)$ (Equation (35)), and then Equation (7) provides

(37) $$\begin{array}{c} P\left(x,t\right)∼\frac{e^{-\frac{t}{\langle \tau \rangle }-\frac{x^{2}}{4D_{+}t}}}{2\sqrt{4\pi D_{+}t}}+\frac{\delta \left(x\right)e^{-\frac{t}{\langle \tau \rangle }}}{2}+\frac{t}{\langle \tau \rangle }\left\{\frac{2e^{-\frac{x^{2}}{4D_{+}t}}}{\sqrt{4\pi D_{+}t}}-\frac{\left|x\right|}{2D_{+}t}\left[1-Erf\left(\frac{\left|x\right|}{\sqrt{4D_{+}t}}\right)\right]\right\}, \end{array}$$

which agrees with the results obtained above in Equation (26), when ${\langle \tau \rangle }_{+}={\langle \tau \rangle }_{-}$ and for t⟶0, since, in that limit, exp(−t/〈τ〉)∼1−t/〈τ〉. Particularly for x≠0 and taking x⟶0, Equation (37) yields a tent shaped propagator described by

(38) $$\begin{array}{c} P\left(x,t\right)∼\frac{3t+\langle \tau \rangle }{4\langle \tau \rangle \sqrt{\pi D_{+}t}}-\frac{\left|x\right|}{2D_{+}\langle \tau \rangle }+K_{2}x^{2}, \end{array}$$

with $K_{2}=\left(5t-\langle \tau \rangle \right)/\left[16\langle \tau \rangle \sqrt{\pi }{\left(D_{+}t\right)}^{\frac{3}{2}}\right]$ and in concordance with Equation (27). On the other hand, within this short time limit, for large displacements x⟶∞, the two terms between curly braces in Equation (37) cancel each other, and only the first term in Equation (37) is left (when x≠0). This is due to the expansion of $1-Erf\left(z\right)∼\exp\left(-z^{2}\right)/\left(\sqrt{\pi }z\right)$ for z⟶∞, in our case $z=\left|x\right|/\sqrt{4D_{+}t}$. Then, Equation (37) can be approximated by

(39) $$\begin{array}{c} P\left(x,t\right)\underset{x\to \infty }{∼}\frac{e^{-\frac{t}{\langle \tau \rangle }-\frac{x^{2}}{4D_{+}t}}}{2\sqrt{4\pi D_{+}t}}. \end{array}$$

This Gaussian behavior of P(x,t) at the tails is expected. The large |x| limit is dominated by trajectories for which no transitions to $D_{-}$ were performed and a pure diffusion process with $D_{+}$ occurs.

When t>>〈τ〉, ergodicity is satisfied and, therefore, the system on average visits the two states the same amount of time. Namely, the *ensemble* average of $p_{+}$ is equal to the corresponding fraction of the average waiting times. In this case, when ${\langle \tau \rangle }_{+}={\langle \tau \rangle }_{-}$, the occupation fraction is concentrated at $p_{+}=1/2$. Thus, the PDF of $p_{+}$ is represented by the delta function

(40) $$\begin{array}{c} g_{t}\left(p_{+}\right)\overset{}{\underset{t\to \infty }{\to }}\delta \left(p_{+}-\frac{1}{2}\right). \end{array}$$

Substituting Equation (40) in Equation (7), we recover Gaussian statistics for the displacements

(41) $$\begin{array}{c} P\left(x,t\right)∼\frac{e^{-\frac{x^{2}}{2D_{+}t}}}{\sqrt{2\pi D_{+}t}}. \end{array}$$

**Figure 5:**
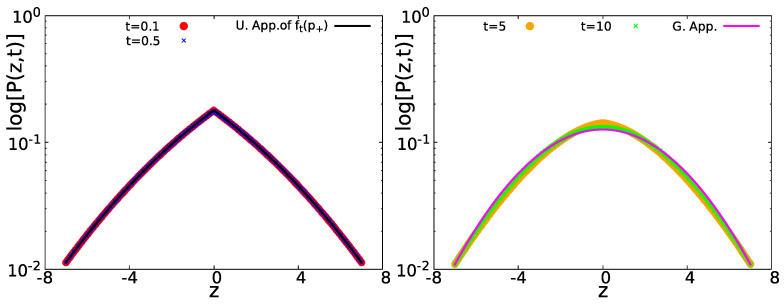
P(z,t) in semi-log scale, with $z=x/\sqrt{t}$. Left: For short times t=0.1 (red circles) and t=0.5 (blue crosses), P(z,t) is represented by Equation (37) (black solid line) with a tent like shape. Right: The same for large times t=5 (orange circles) and t=10 (green crosses), P(z,t) converges to the Gaussian distribution Equation (41) (magenta solid line). In all the cases, $D_{+}=10$, $D_{-}=0$, and 〈τ〉=1 were used.

In Figure 5, we present the two different limit distributions for P(x,t) in the short time limit t=0.1 (red circles) and t=0.5 (blue crosses) Equation (37) and the Gaussian limit for t=5 (orange circles) and t=10 (green crosses) Equation (41), for the normalized variable $z=x/\sqrt{t}$. As we can see, the displacements for short times follow a tent shape (black solid line) and a Gaussian one in the long time limit (magenta solid line).

#### 2.3.2. Different Mean Waiting Times ${\langle \tau \rangle }_{+}\neq {\langle \tau \rangle }_{-}$

Relaxing the assumption of equal mean waiting times for exponentially distributed sojourn times in the model, we have that ${\langle \tau \rangle }_{+}\neq {\langle \tau \rangle }_{-}$, with waiting times following Equation (31). As mentioned, for equilibrium initial conditions, the PDF of $T_{+}$ is given by Equation (3). Let ${\hat{f}}_{s}^{\pm }\left(u\right)$ be the double Laplace transform of $f_{t}^{\pm }\left(T_{+}\right)$, defined as ${\hat{f}}_{s}^{\pm }\left(u\right)=\int_{0}^{\infty }\int_{0}^{\infty }f_{t}\left(T_{+}\right)\exp\left(-uT_{+}-st\right)\;dT_{+}\;dt$. Then, the different terms of the PDF of $T_{+}$ in Equation (3) are provided in Laplace space, by [42,47,49]

(42) $$\begin{array}{ccc} {\hat{f}}_{s}^{+}\left(u\right) & = & \left\{{\hat{\psi }}_{+}\left(s+u\right)\left[\frac{1-{\hat{\psi }}_{-}\left(s\right)}{s}\right]+\frac{1-{\hat{\psi }}_{+}\left(s+u\right)}{s+u}\right\}\frac{1}{1-{\hat{\psi }}_{+}\left(s+u\right){\hat{\psi }}_{-}\left(s\right)}, \end{array}$$

(43) $$\begin{array}{ccc} {\hat{f}}_{s}^{-}\left(u\right) & = & \left\{{\hat{\psi }}_{-}\left(s\right)\left[\frac{1-{\hat{\psi }}_{+}\left(s+u\right)}{s+u}\right]+\frac{1-{\hat{\psi }}_{-}\left(s\right)}{s}\right\}\frac{1}{1-{\hat{\psi }}_{+}\left(s+u\right){\hat{\psi }}_{-}\left(s\right)}. \end{array}$$

Summing up Equations (42) and (43) according to Equation (3), we obtain, for exponentially distributed waiting times,

(44) $$\begin{array}{c} {\hat{f}}_{s}\left(u\right)=\frac{{\langle \tau \rangle }_{-}^{2}+{\langle \tau \rangle }_{+}^{2}\left(1+{\langle \tau \rangle }_{-}s\right)+{\langle \tau \rangle }_{+}{\langle \tau \rangle }_{-}\left[2+{\langle \tau \rangle }_{-}\left(s+u\right)\right]}{\left({\langle \tau \rangle }_{+}+{\langle \tau \rangle }_{-}\right)\left[{\langle \tau \rangle }_{-}s+{\langle \tau \rangle }_{+}\left(1+{\langle \tau \rangle }_{-}s\right)\left(s+u\right)\right]}. \end{array}$$

Taking the double inverse Laplace transform of Equation (44) with respect to $u\Leftrightarrow T_{+}$ and s⇔t and changing variables to $p_{+}=T_{+}/t$, we obtain the PDF for $p_{+}$ (see details in Appendix F)

(45) $$\begin{array}{cc} & g_{t}\left(p_{+}\right)=\frac{{\langle \tau \rangle }_{-}e^{-\frac{t}{{\langle \tau \rangle }_{-}}}}{{\langle \tau \rangle }_{+}+{\langle \tau \rangle }_{-}}\delta \left(p_{+}\right)+\frac{{\langle \tau \rangle }_{+}e^{-\frac{t}{{\langle \tau \rangle }_{+}}}}{{\langle \tau \rangle }_{+}+{\langle \tau \rangle }_{-}}\delta \left(1-p_{+}\right)+\frac{2t}{{\langle \tau \rangle }_{+}+{\langle \tau \rangle }_{-}}\{I_{0}\left(2t\sqrt{\frac{p_{+}\left(1-p_{+}\right)}{{\langle \tau \rangle }_{+}{\langle \tau \rangle }_{-}}}\right)\\ & +\left[\frac{\left(1-p_{+}\right)\sqrt{{\langle \tau \rangle }_{+}{\langle \tau \rangle }_{-}}}{{\langle \tau \rangle }_{+}}+\frac{p_{+}\sqrt{{\langle \tau \rangle }_{+}{\langle \tau \rangle }_{-}}}{{\langle \tau \rangle }_{-}}\right]\frac{I_{1}\left(2t\sqrt{\frac{p_{+}\left(1-p_{+}\right)}{{\langle \tau \rangle }_{+}{\langle \tau \rangle }_{-}}}\right)}{2\sqrt{p_{+}\left(1-p_{+}\right)}}\}e^{-\frac{tp_{+}}{{\langle \tau \rangle }_{+}}-\frac{t(1-p_{+})}{{\langle \tau \rangle }_{-}}}. \end{array}$$

For the case when ${\langle \tau \rangle }_{+}={\langle \tau \rangle }_{-}=\langle \tau \rangle$, Equation (45) recovers Equation (34) obtained by the methods reported in [52,53]. The case of non-equilibrium initial conditions is shown in Appendix D.

In the short time regime, strictly speaking when $t<<{\langle \tau \rangle }_{\pm }$, by expanding Equation (45) for t⟶0, $g_{t}\left(p_{+}\right)$ can be approximated by the uniform distribution

(46) $$\begin{array}{c} g_{t}\left(p_{+}\right)∼\frac{{\langle \tau \rangle }_{-}e^{-\frac{t}{{\langle \tau \rangle }_{-}}}}{{\langle \tau \rangle }_{+}+{\langle \tau \rangle }_{-}}\delta \left(p_{+}\right)+\frac{{\langle \tau \rangle }_{+}e^{-\frac{t}{{\langle \tau \rangle }_{+}}}}{{\langle \tau \rangle }_{+}+{\langle \tau \rangle }_{-}}\delta \left(1-p_{+}\right)+\frac{2t}{{\langle \tau \rangle }_{+}+{\langle \tau \rangle }_{-}}. \end{array}$$

As mentioned above, Equation (25) that was deduced for general PDFs of waiting times, encloses the particular case of Equation (46). For the uniform approximation of $g_{t}\left(p^{+}\right)$ (Equation (46)), the positional PDF (Equation (7)) is

(47) $$\begin{array}{ccc} P(x,t) & ∼ & \frac{{\langle \tau \rangle }_{+}e^{-\frac{t}{{\langle \tau \rangle }_{+}}-\frac{x^{2}}{4D_{+}t}}}{\left({\langle \tau \rangle }_{+}+{\langle \tau \rangle }_{-}\right)\sqrt{4\pi D_{+}t}}+\frac{{\langle \tau \rangle }_{-}}{{\langle \tau \rangle }_{+}+{\langle \tau \rangle }_{-}}e^{-\frac{t}{{\langle \tau \rangle }_{-}}}\delta \left(x\right)\\ \; & + & \frac{2te^{-\frac{x^{2}}{4D_{+}t}}}{\left({\langle \tau \rangle }_{+}+{\langle \tau \rangle }_{-}\right)\sqrt{\pi D_{+}t}}-\frac{\left|x\right|}{D_{+}\left({\langle \tau \rangle }_{+}+{\langle \tau \rangle }_{-}\right)}\left[1-Erf\left(\frac{\left|x\right|}{\sqrt{4D_{+}t}}\right)\right], \end{array}$$

which agrees with the general case described by Equation (26).

Similar to Section 2.2, in the limit t⟶∞, the PDF of the occupation fraction $g_{t}\left(p_{+}\right)$ follows Equation (28). In addition, the PDF of the displacements in the long time regime is given by Equations (7) and (29), recovering Gaussianity.

**Figure 6:**
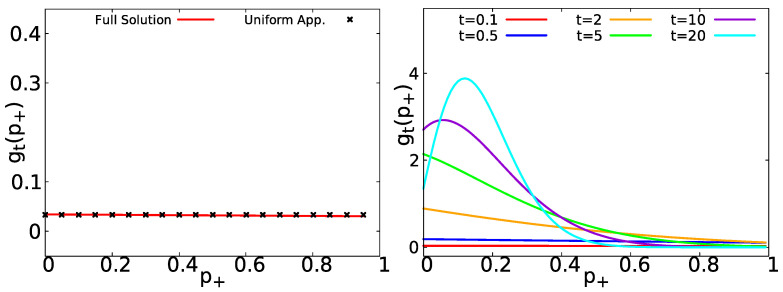
Left: Comparison between $g_{t}\left(p_{+}\right)$ Equation (45) (red solid line) and the uniform approximation Equation (46) (black asterisks) for ${\langle \tau \rangle }_{+}=1$, ${\langle \tau \rangle }_{-}=5$ and t=0.1. Right: $g_{t}\left(p_{+}\right)$ Equation (45) for ${\langle \tau \rangle }_{+}=1$, ${\langle \tau \rangle }_{-}=5$ and t∈{0.1,0.5,2,5,10,20}.

In Figure 6, we show $g_{t}\left(p_{+}\right)$ for exponential waiting times with ${\langle \tau \rangle }_{+}=1$ and ${\langle \tau \rangle }_{-}=5$, in the left panel, we compare the uniform approximation of Equation (46) (black asterisks) with the full solution Equation (45) (red solid line), observing an excellent agreement. In the right panel of Figure 6, the behavior of $g_{t}\left(p_{+}\right)$ (as provided by Equation (45)) is displayed. As we can see, it starts with a uniform distribution for short times and then it evolves to a peaked distribution centered at $p_{+}={\langle \tau \rangle }_{+}/\left({\langle \tau \rangle }_{+}+{\langle \tau \rangle }_{-}\right)=1/6$. As shown in Appendix D, for non-equilibrium initial condition, the PDF of $p_{+}$ is still uniform within the short time regime. See also Appendix B for other similar cases.

**Figure 7:**
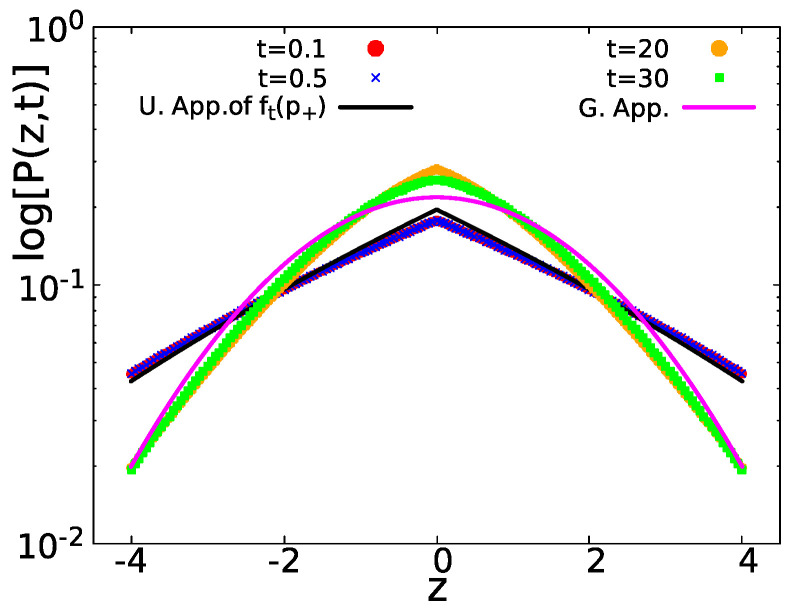
For a system with, ${\langle \tau \rangle }_{+}=1$ and ${\langle \tau \rangle }_{-}=5$, P(z,t) in semi-log scale, with $z=x/\sqrt{t}$. For short times t=0.1 (red circles) and t=0.5 (blue crosses), P(z,t) is represented by Equation (47) (black solid line) with a tent like shape. For large times t=20 (orange circles) and t=30 (green diamonds), P(z,t) converges to the Gaussian statistics Equation (29) (magenta solid line). In all the cases, $D_{+}=10$ and $D_{-}=0$ were used. Compared with Figure 5, in this case, the Gaussian curve is above the tent curve, contrary to the case with equal mean waiting times. This is because the coefficient of the Gaussian curve Equation (29) is bigger compared with the weight of the delta peak in Equation (47). In Figure 5, we have the opposite, and the weight of the corresponding delta function in Equation (37) is bigger compared with the Gaussian Equation (41).

Finally, in Figure 7, we show the corresponding positional spreading for the normalized variable $z=x/\sqrt{t}$. As we can see in the short time, t=0.1 (red circles) and t=0.5 (blue crosses) P(z,t) (given by Equation (47)) attain a tent-like shape. In the long run, t=20 (orange circles) and t=30 (green squares) P(z,t) have a Gaussian distribution given by Equation (29).

## 3. Discussion

### 3.1. The Histogram of the Diffusion Coefficient as Extracted from Experimental Data

#### 3.1.1. Super-Statistics

We have found that at x=0, P(x,t) exhibits a cusp. A mathematically similar non-analytical behavior is found using an approach called super-statistics [12,14,21,54], which was used to explain laboratory observations. This framework postulates that the distribution of diffusion constants in the system is exponential, namely P(D)=exp(−D/〈D〉)/〈D〉 for D>0 and 〈D〉 the average diffusivity. Then, the diffusion follows a Gaussian process with a random *D*. This approach gives

(48) $$\begin{array}{c} P\left(x,t\right)=\int_{0}^{\infty }\frac{e^{-\frac{x^{2}}{4Dt}}}{\sqrt{4\pi Dt}}\frac{e^{-\frac{D}{\langle D\rangle }}}{\langle D\rangle }dD=\frac{e^{-\frac{\left|x\right|}{\langle D\rangle t}}}{4\langle D\rangle t}. \end{array}$$

Here, on the right-hand side, we have the Laplace PDF, which was used by Laplace in 1774 [55] to describe his linear law of errors [56]. In addition, as in our case, within the super-statistics method, we see in Equation (48) a non-analytical behavior since $P\left(x,t\right)∼C_{1}-C_{2}\left|x\right|$, for small *x* and with $C_{1},C_{2}$ constants. Our work does not support the Laplace law, see Equations (26) and (27). However, maybe more importantly, the whole approach presented in this manuscript differs from the super-statistical approach in the following way. In our model, we have two diffusion constants, $D_{+}$ and $D_{-}=0$ (see Appendix A for the case when $D_{-}\neq 0$). Hence, the PDF of diffusion constants is $P\left(D\right)=a\delta \left(D\right)+b\delta \left(D-D_{+}\right)$, with a,b≥0. It follows that the super-statistical approach predicts that the diffusing packet P(x,t) is a sum of a delta function corresponding to non-moving particles and a Gaussian packet describing the movers. Thus, when the non-moving particles are excluded, we have perfect Gaussian behavior. This is actually correct, to leading order, for very short times. Thus, the super-statistical approach gives the correct t⟶0 behavior but fails to predict the main issue (in our opinion), and that is the cusp on x=0. To explore the non-analytical behavior, one needs to go to the next order terms in the expansion to include paths with a transition between states. Then, as we have shown, the equilibrium initial condition yields a uniform distribution of the occupation fraction Equation (25). It is this fact that brings the non-analytical behavior in the final result for P(x,t) Equation (27), graphically represented by a “tent” see Figure 3, Figure 5, and Figure 7. It follows that the exponential conspiracy in which distribution of diffusion constants is exponential is not a necessary condition for a cusp like behavior of P(x,t). We further remark that the non-analytical behavior is found also in the context of normal diffusion in [35,36,37] and within the anomalous one at [31,32,33,34,36,38,39,40].

#### 3.1.2. Time Average MSD

We note that, in single molecule experiments, the time average mean squared displacement (TAMSD) is used in many cases to estimate the distribution of diffusion constants [2,5,6]. Since time averages are recorded over a finite measurement time, the time average fluctuates. Hence, we have naturally a distribution of the estimator for the diffusion parameters. In addition, the aforementioned two delta peak distribution of *D*, i.e., on $D_{+}$ and on $D_{-}$, is expected to be smeared out. This topic was extensively studied in a wide variety of models [57,58].

We now investigate the fluctuations of the time averaged diffusivities in a two state model and their implications in the distribution of diffusion coefficients obtained from real experimental data. For a further analysis of the time average diffusivity within a two state system, see [59,60].

**Figure 8:**
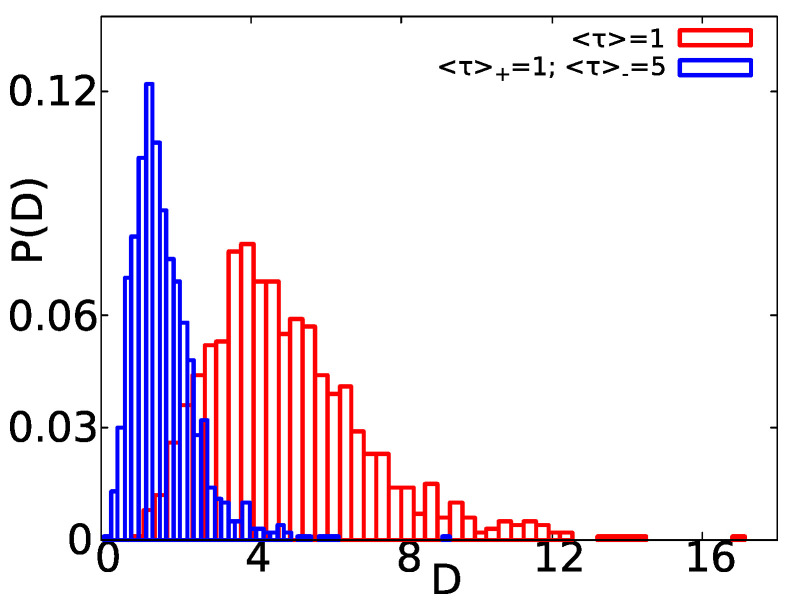
Distribution of diffusion coefficients P(D) obtained via TAMSD analysis of simulated trajectories of a two state system with $D_{+}=10$, $D_{-}=0$ and exponentially distributed waiting times. From the linear plots of the TAMSD versus the lag time estimates of *D* were extracted. We show two cases, the first for a system with the same mean waiting times ${\langle \tau \rangle }_{+}={\langle \tau \rangle }_{-}=\langle \tau \rangle =1$ (red boxes). In addition, the PDF of *D* for a system with different mean waiting times with ${\langle \tau \rangle }_{+}=1$ and ${\langle \tau \rangle }_{-}=5$ is also shown (blue boxes). For the system with the same mean waiting times, the average diffusivity found in the simulations is 〈D〉=4.98, and, for the case of different mean waiting times, we have 〈D〉=1.69. In both cases, we used t=1000 and 1000 trajectories.

We note that, in different single particle tracking experiments with non-Gaussian propagators, the recorded distribution of the diffusion coefficient *D* (obtained by means of TAMSD analysis) is relatively broad and peaked close to the origin [2,5,6]. Those experimental distributions of *D* are typically fitted by exponential [6] or gamma [2] distributions. Within the two state model, the diffusivity takes only two possible values $D_{-}$ or $D_{+}$, but the respective TAMSD analysis gives values of *D* around $D_{-}$ and $D_{+}$ [59]. The average *D* is given by $\langle D\rangle =\left(D_{+}{\langle \tau \rangle }_{+}+D_{-}{\langle \tau \rangle }_{-}\right)/\left({\langle \tau \rangle }_{+}+{\langle \tau \rangle }_{-}\right)$. Thus, how different is the distribution of the diffusivities, extracted via TAMSD techniques, in a two state model compared with the one present in single molecule experiments? As we show next, this will be determined by the values of $D_{\pm }$ and ${\langle \tau \rangle }_{\pm }$. In Figure 8, we show the distribution of the diffusion coefficients obtained by means of TAMSD analysis for $D_{+}=10$, $D_{-}=0$ and exponentially distributed waiting times. We show two different cases, the first one with the same mean waiting times ${\langle \tau \rangle }_{+}={\langle \tau \rangle }_{-}=1$ (see red boxes). In addition, the second one with different mean waiting times, such that ${\langle \tau \rangle }_{+}=1$ and ${\langle \tau \rangle }_{-}=5$ (see blue boxes).

As we can see in Figure 8, when the difference between the diffusion coefficients is large, as in our case $D_{+}=10>D_{-}=0$, P(D) is relatively broad. Nonetheless, for the case with ${\langle \tau \rangle }_{+}=1$ and ${\langle \tau \rangle }_{-}=5$, the peak of P(D) is closer to the origin compared to the case with 〈τ〉=1.

This difference between mean waiting times in each state is the second factor that determines the shape of P(D). For instance, when this difference is such that ${\langle \tau \rangle }_{+}<{\langle \tau \rangle }_{-}$, it is straightforward that the more the process spends in the state “−”, the more the observed values of *D* will be closer to $D_{-}$. In this latter case, the distribution of *D* is peaked close to the origin since $D_{-}<D_{+}$. Thus, we can say that, when the differences between the diffusivities (and the mean waiting times) in the different states are pronounced, i.e., $D_{-}<<D_{+}$ and ${\langle \tau \rangle }_{+}<<{\langle \tau \rangle }_{-}$, P(D) in the two sate model resembles the distributions found in single molecule experiments [2,5,6].

## 4. Conclusions

From symmetry of the density of spreading particles P(x,t)=P(−x,t), we expect an analytical expansion of the propagator as $P\left(x,t\right)∼K_{1}-K_{2}x^{2}+\ldots$, with $K_{1},K_{2}$ constants. Instead, in the two state model handled throughout this work, we get an expansion that is linear in |x|, see Equation (27). This is a non-analytical expansion graphically represented by a tent like structure, see Figure 3, Figure 5, and Figure 7. As mentioned above, Laplace in 1774 considered a similar non-analytical PDF, P(x)=exp(−|x|)/2 for −∞<x<∞ [55,56]. However, the expression we find is clearly non-exponential, see Equation (26). Furthermore, for large *x*, we get a Gaussian behavior for P(x,t). It should be noted that a non-analytical behavior is found only if $D_{-}=0$, see Appendix A for further details. In practice, we may approach the non-analytical features of P(x,t), as $D_{-}$ is getting small.

Recently, a very general theory was developed for the non-Gaussian spreading of packets of particles. Using a CTRW framework, it was shown that, for any analytical PDF of waiting times, for large *x* limit P(x,t)∼exp(−C|x|ln|x|), with *C* a constant [22]. In the former model, we thus find exponential tails for large *x*, while, here, the anomaly, i.e., the cusp or tent like feature of P(x,t), comes from the small *x* limit.

Recently, Postnikov et al. [37] investigated a model of diffusion in a quenched disordered setting, where the diffusive field is spatially varying. They showed that equilibrium initial conditions play a major role stating: “within the class of models with quenched disorder, the Itô model under equilibrium conditions is the only promising candidate for the description of Brownian Non Gaussian diffusion (BnG).” Note that here the definition of BnG means a model or system where the MSD is increasing linearly *for all times* and the propagator is non-Gaussian. Our model uses a time dependent diffusivity, and we showed that equilibrium initial conditions are indeed a key requirement. Here, we note that BnG does not imply a cusp, and vice versa. Namely, we may find a system where the MSD is increasing linearly in time, for the entire span of time, with or without a cusp for P(x,t) at x=0. The main focus of our work is the presence of a cusp for P(x,t). Regarding the behavior of the MSD, it can be shown that, when equilibrium initial conditions are applied, $\langle T_{+}\rangle =\left({\langle \tau \rangle }_{+}t\right)/\left[{\langle \tau \rangle }_{+}+{\langle \tau \rangle }_{-}\right]$, for all times *t* (see Appendix G). Then, by Equations (A62) and (A68), the MSD is provided by

(49) $$\langle x^{2}\left(t\right)\rangle =\left(\frac{D_{+}{\langle \tau \rangle }_{+}+D_{-}{\langle \tau \rangle }_{-}}{{\langle \tau \rangle }_{+}+{\langle \tau \rangle }_{-}}\right)t,$$

for any time *t*. Thus, if the process starts from equilibrium, the MSD grows linearly for all times and we have BnG. Nevertheless, we would like to emphasize that our model is exhibiting BnG, but specifically P(x,t) has a cusp only if $D_{-}=0$, and practically when $D_{-}<<D_{+}$.

To summarize, we emphasize that we have shown, by means of the statistics of the temporal occupation, that there is a universality for the PDF of the temporal occupation fraction in a two state model. For PDFs of waiting times with finite first moments, $g_{t}\left(p_{+}\right)$ can be approximated by a uniform distribution following Equation (25). This leads to tent like decaying propagators (Equation (26)) similar to those found in many experimental systems. We corroborate our results by solving analytically a two state system with exponentially distributed waiting times. We have shown that, either for short or long times, the distribution of displacements P(x,t) has a general form, either a “tent” or a Gaussian bell curve. These two endpoints of the positional PDF are independent of the actual form of the distribution of waiting times. The crucial point within our framework is the generality of the behavior of the PDF of the occupation fraction $p_{+}$, being a uniform distribution for short times and a delta peak for long times. The former was found for a system with equilibrium initial conditions. We note that, for certain types of non-equilibrium initial conditions, we can still get a uniform PDF for the fraction occupation time; however, this is not generic (see details in Appendix B). Therefore, the non-Gaussian features are readily present in our model within the short time regime, and regardless of the specifics of the waiting times.

Mathematically, we presented an expansion in terms of the number of transitions from state + to − and backwards. Naturally, for very short times, the leading contribution to the packet comes from the paths with zero transitions, and then the packet is simply a sum of two Gaussian curves with diffusion coefficients $D_{+}$ and $D_{-}$. However, we showed that, by going to next order terms in the expansion, namely considering the paths with a single jump, we get the cusp like shape, found in the limit $D_{-}\to 0$. Thus, the whole effect is achieved by using a perturbation approach obtaining the leading order correction to the trivial behavior. Put differently, a widely popular super statistical approach is found to miss one of the main issues of the field, namely the cusp in P(x,t). A super-statistical approach [14,54] uses a distribution of diffusivities, which in our model is a sum of two delta functions, at $D_{-}=0$ and $D_{+}$. This does not give the cusp, as it is merely the zeroth order of the perturbation theory developed here.

## Figures and Tables

**Figure A1:**
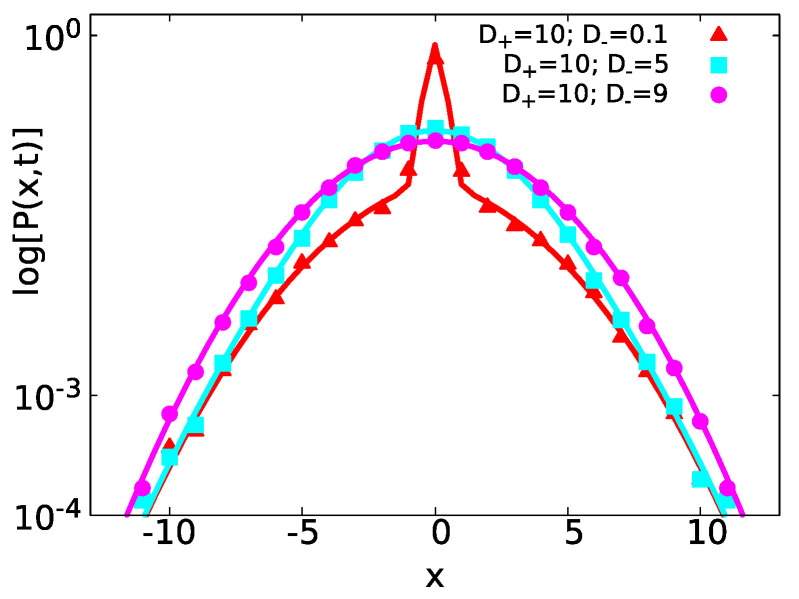
Distribution of displacements P(x,t) obtained by simulations of a two state system with $D_{+}>D_{-}>0$ and gamma distributed waiting times τ∼Gamma(3,1) at $D_{+}$ and τ∼Gamma(6,1) at $D_{-}$ following Equation (30). We compare with Equation (A4) (solid lines) with t=0.5, ${\langle \tau \rangle }_{+}=3$, ${\langle \tau \rangle }_{-}=6$, $D_{+}=10$. For $D_{-}=0.1$ (red triangles), $D_{-}=5$ (cyan squares) and $D_{-}=9$ (magenta circles). Exponential like decaying is present at small values for *x*, when $D_{+}=10>>D_{-}=0.1$ (red solid line). In the cases when $D_{-}⟶D_{-}$ (cyan and magenta solid lines), P(x,t) follows a full Gaussian distribution.

**Figure A3:**
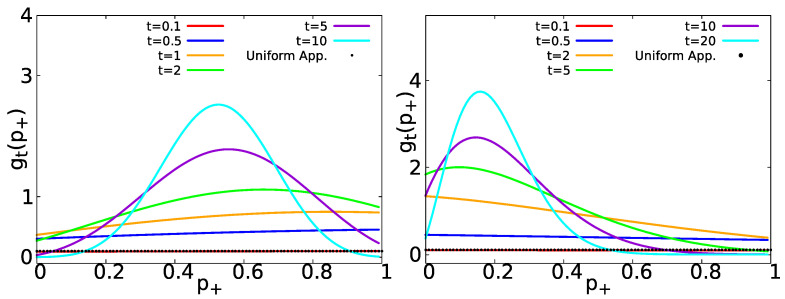
Left: $g_{t}\left(p_{+}\right)$ Equation (A34) for 〈τ〉=1 and t∈{0.1,0.5,1,2,5,10} and non-equilibrium initial conditions (starting from state “+”). The uniform approximation of $g_{t}\left(p_{+}\right)$ Equation (A35) for t=0.1 is shown in black circles. Right: $g_{t}\left(p_{+}\right)$ Equation (A39) for ${\langle \tau \rangle }_{+}=1$, ${\langle \tau \rangle }_{-}=5$ and t∈{0.1,0.5,2,5,10,20} and non-equilibrium initial conditions (starting from state “+”). The uniform approximation of $g_{t}\left(p_{+}\right)$ Equation (A40) for t=0.1 is shown in black circles.

## Data Availability

All the data sets obtained by numerical simulations or data analysis are available from the corresponding authors upon request.

## Abbreviations

The following abbreviations are used in this manuscript:

CTRW	Continuous Time Random Walk
TAMSD	Time Average Mean Squared Displacement

## Appendix A. A Two State Model with $D_{+}>D_{-}>0$

When $D_{+}>D_{-}>0$, the process for the displacements becomes

(A1) $$\begin{array}{c} x\left(t\right)=\sqrt{2D_{+}T_{+}}\xi_{1}+\sqrt{2D_{-}\left(t-T_{+}\right)}\xi_{2}, \end{array}$$

with $\xi_{1}$ and $\xi_{2}$ each i.i.d. Gaussian variables. In this case, the form of the conditioned PDF is given by

(A2) $$\begin{array}{c} P\left(x,t|T_{+}\right)=\frac{e^{-\frac{x^{2}}{4[D_{+}T_{+}+D_{-}\left(t-T_{+}\right)]}}}{\sqrt{4\pi [D_{+}T_{+}+D_{-}\left(t-T_{+}\right)]}}. \end{array}$$

Then, the marginal distribution for the displacements follows

(A3) $$\begin{array}{c} P\left(x,t\right)=\int_{0}^{1}\frac{e^{-\frac{x^{2}}{4t[D_{+}p_{+}+D_{-}\left(1-p_{+}\right)]}}}{\sqrt{4\pi t[D_{+}p_{+}+D_{-}\left(1-p_{+}\right)]}}g_{t}\left(p_{+}\right)dp_{+}. \end{array}$$

### Appendix A.1. P(x,t) for Arbitrary Waiting Times

As we did in Section 2.2.1.1, using the general forms obtained above, i.e., Equations (9), (17), (18), (23), and (28), we can analyze P(x,t) in the short and long time limits.

#### Appendix A.1.1. Short Time Regime

Substituting Equation (25) in Equation (A3), we get

(A4) $$\begin{array}{c} \begin{array}{ccc} P(x,t) & = & \frac{{\langle \tau \rangle }_{+}}{{\langle \tau \rangle }_{+}+{\langle \tau \rangle }_{-}}\left(1-\frac{t}{{\langle \tau \rangle }_{+}}\right)\frac{e^{-\frac{x^{2}}{4D_{+}t}}}{\sqrt{4\pi D_{+}t}}+\frac{{\langle \tau \rangle }_{-}}{{\langle \tau \rangle }_{+}+{\langle \tau \rangle }_{-}}\left(1-\frac{t}{{\langle \tau \rangle }_{-}}\right)\frac{e^{-\frac{x^{2}}{4D_{-}t}}}{\sqrt{4\pi D_{-}t}}\\ & + & \frac{2}{\sqrt{\pi }\left({\langle \tau \rangle }_{+}+{\langle \tau \rangle }_{-}\right)\left[D_{-}-D_{+}\right]}\{\sqrt{D_{-}t}e^{-\frac{x^{2}}{4D_{-}t}}-\sqrt{D_{+}t}e^{-\frac{x^{2}}{4D_{+}t}}\\ & - & \frac{\sqrt{\pi }\left|x\right|}{2}\left[Erf\left(\frac{\left|x\right|}{\sqrt{4D_{+}t}}\right)-Erf\left(\frac{\left|x\right|}{\sqrt{4D_{-}t}}\right)\right]\}. \end{array} \end{array}$$

In Figure A1, we compare Equation (A4) (in solid lines) and P(x,t) obtained by simulations of a two state model for a fixed value of $D_{+}$ and different values of $D_{-}$, such that $D_{+}>D_{-}$. In all cases, we used gamma distributed waiting times τ∼Gamma(3,1) for the state with $D_{+}$ and τ∼Gamma(6,1) for the state with $D_{-}$ (the gamma distribution is defined by Equation (30)). As we can see when $D_{+}>>D_{-}$, e.g., $D_{+}=10$ and $D_{-}0.1$ (red triangles), the PDF of the displacements at small values of *x* has a non-Gaussian peak; thereafter, for large values of *x*, it follows a Gaussian distribution. When the values of $D_{-}$ approach $D_{+}$ (cyan squares and magenta circles), P(x,t) is fully described by Gaussian statistics even in the short time limit.

#### Appendix A.1.2. Long Time Regime

In the long time limit, the PDF of temporal occupation fraction is provided by Equation (28), then, according to Equation (A3), the PDF of the displacements is determined by

(A5) $$\begin{array}{c} P\left(x,t\right)∼\sqrt{\frac{{\langle \tau \rangle }_{+}+{\langle \tau \rangle }_{-}}{4\pi t[D_{+}{\langle \tau \rangle }_{+}+D_{-}{\langle \tau \rangle }_{-}]}}e^{-\frac{x^{2}\left({\langle \tau \rangle }_{+}+{\langle \tau \rangle }_{-}\right)}{4t[D_{+}{\langle \tau \rangle }_{+}+D_{-}{\langle \tau \rangle }_{-}]}}. \end{array}$$

Thus, the Gaussian limit is also restored.

### Appendix A.2. P(x,t) for Exponentially Distributed Waiting Times with ${\langle \tau \rangle }_{+}\neq {\langle \tau \rangle }_{-}$

In the short time regime, we can use the uniform approximation Equation (46) in Equation (A3), and the distribution for the displacements yields

(A6) $$\begin{array}{ccc} P(x,t) & ∼ & \frac{{\langle \tau \rangle }_{+}}{{\langle \tau \rangle }_{+}+{\langle \tau \rangle }_{-}}\frac{e^{-\frac{t}{{\langle \tau \rangle }_{+}}-\frac{x^{2}}{4D_{+}t}}}{\sqrt{4\pi D_{+}t}}+\frac{{\langle \tau \rangle }_{-}}{{\langle \tau \rangle }_{+}+{\langle \tau \rangle }_{-}}\frac{e^{-\frac{t}{{\langle \tau \rangle }_{-}}-\frac{x^{2}}{4D_{-}t}}}{\sqrt{4\pi D_{-}t}}\\ \; & + & \frac{1}{\left({\langle \tau \rangle }_{+}+{\langle \tau \rangle }_{-}\right)\left(D_{-}-D_{+}\right)\sqrt{\pi }}\{\sqrt{4D_{-}t}e^{-\frac{x^{2}}{4D_{-}t}}-\sqrt{4D_{+}t}e^{-\frac{x^{2}}{4D_{+}t}}\\ \; & + & \pi \left[Erf\left(\frac{\left|x\right|}{\sqrt{4D_{-}t}}\right)-Erf\left(\frac{\left|x\right|}{\sqrt{4D_{+}t}}\right)\right]\}. \end{array}$$

For the long time regime, we use $g_{t}\left(p_{+}\right)$ provided by Equation (28); then, according to Equation (A3), the PDF of the displacements follows Gaussian statistics described by Equation (A5).

## Appendix B. A Complementary Deduction of $f_{t}^{\pm }\left(T_{+}|1\right)$

In this section, we obtain Equation (23) from the definition of conditional probability. The conditional probability of $T_{+}$, given that *N* jumps have been made, follows

(A7) $$\begin{array}{c} f_{t}^{\pm }\left(T_{+}|N\right)=\frac{f_{t}^{\pm }\left(T_{+},N\right)}{Q_{t}^{\pm }\left(N\right)}, \end{array}$$

with the distribution of jumps defined by [46]

(A8) $$\begin{array}{c} Q_{t}^{\pm }\left(N\right)=\langle 1_{\left(t_{N},t_{N+1}\right)}\left(t\right)\rangle , \end{array}$$

with $1_{\left(a,b\right)}\left(t\right)$ the indicator function, such that it is equal to one if t∈(a,b) and zero if t∉(a,b). The average 〈·〉 is over all the the values of $\tau_{i}$’s, with i∈{1,2,…,N+1}.In addition, $f_{t}^{\pm }\left(T_{+},N\right)$ is the joint probability of $T_{+}$ and *N*, which satisfies [46]

(A9) $$\begin{array}{c} f_{t}^{\pm }\left(T_{+},N\right)=\langle \delta \left(y-T_{+}\right)1_{\left(t_{N},t_{N+1}\right)}\left(t\right)\rangle , \end{array}$$

with $t_{N}=\tau_{1}+\ldots +\tau_{N}$ and the average 〈·〉 defined as above.

Let us find Equations (A8) and (A9) and therefore Equation (A7) for the case N=1. It is important to notice that, for the case of equilibrium initial conditions such as the one handled in Section 2.2, when N=1, the corresponding average on $\tau_{1}$ is given by the forward recurrence distribution Equation (10). Following Equation (A8), and taking the Laplace transform defined as ${\hat{Q}}_{s}^{\pm }\left(N\right)=\int_{0}^{\infty }e^{-st}Q_{t}^{\pm }\left(N\right)dt$, after simple manipulations, we obtain

(A10) $$\begin{array}{ccc} {\hat{Q}}_{s}^{\pm }\left(1\right) & = & \int_{0}^{\infty }e^{-s\tau_{1}}f_{eq}^{\pm }\left(\tau_{1}\right)d\tau_{1}\int_{0}^{\infty }\left(\frac{1-e^{-s\tau_{2}}}{s}\right)\psi_{\mp }\left(\tau_{2}\right)d\tau_{2},\\ & = & \left(\frac{1-{\hat{\psi }}_{\pm }\left(s\right)}{{\langle \tau \rangle }_{\pm }s}\right)\left(\frac{1-{\hat{\psi }}_{\mp }\left(s\right)}{s}\right), \end{array}$$

which is already the result shown in Equation (11).

For the joint distribution $f_{t}^{\pm }\left(T_{+},1\right)$, following Equation (A9) and taking the double Laplace transform defined as ${\hat{f}}_{s}^{\pm }\left(u,N\right)=\int_{0}^{\infty }e^{-uT_{+}}\int_{0}^{\infty }e^{-st}f_{t}^{\pm }\left(T_{+},N\right)dtdT_{+}$, after performing the corresponding integrals in the case we started from “+”, we get

(A11) $$\begin{array}{ccc} {\hat{f}}_{s}^{+}\left(u,1\right) & = & \int_{0}^{\infty }e^{-\left(s+u\right)\tau_{1}}f_{eq}^{+}\left(\tau_{1}\right)d\tau_{1}\int_{0}^{\infty }\left(\frac{1-e^{-s\tau_{2}}}{s}\right)\psi_{-}\left(\tau_{2}\right)d\tau_{2},\\ & = & \left(\frac{1-{\hat{\psi }}_{+}\left(s+u\right)}{{\langle \tau \rangle }_{+}\left(s+u\right)}\right)\left(\frac{1-{\hat{\psi }}_{-}\left(s\right)}{s}\right). \end{array}$$

Following the same procedure for the case when the process started from “−”, we obtain

(A12) $$\begin{array}{c} {\hat{f}}_{s}^{-}\left(u,1\right)=\left(\frac{1-{\hat{\psi }}_{-}\left(s\right)}{{\langle \tau \rangle }_{-}\left(s\right)}\right)\left(\frac{1-{\hat{\psi }}_{+}\left(s+u\right)}{s+u}\right). \end{array}$$

Next, we show the connection of the joint PDf Equation (A9) with the uniform distribution of the occupation times Equation (24). Let ${\hat{f}}_{s}^{eq}\left(u,1\right)$ be the double Laplace transform of $f_{t}^{eq}\left(T_{+},1\right)$, i.e., the joint PDF of $T_{+}$ and one single jump, starting from equilibrium. Clearly, the former follows

(A13) $$\begin{array}{c} {\hat{f}}_{s}^{eq}\left(u,1\right)=\frac{{\langle \tau \rangle }_{+}}{{\langle \tau \rangle }_{+}+{\langle \tau \rangle }_{-}}{\hat{f}}_{s}^{+}\left(u,1\right)+\frac{{\langle \tau \rangle }_{-}}{{\langle \tau \rangle }_{+}+{\langle \tau \rangle }_{-}}{\hat{f}}_{s}^{-}\left(u,1\right). \end{array}$$

Using Equations (A11) and (A12) in Equation (A13), we get

(A14) $$\begin{array}{c} {\hat{f}}_{s}^{eq}\left(u,1\right)=\frac{2}{{\langle \tau \rangle }_{+}+{\langle \tau \rangle }_{-}}\left(\frac{1-{\hat{\psi }}_{+}\left(s+u\right)}{s+u}\right)\left(\frac{1-\hat{\psi }\left(s\right)}{s}\right). \end{array}$$

One of the key features of our paper is found when we consider both *u* and *s* to be large. This corresponds to the short time limit, when $T_{+}$ and *t* are of the same order. Then, we may use ${\hat{\psi }}_{+}\left(s+u\right),{\hat{\psi }}_{-}\left(s\right)⟶0$ in Equation (A14), yielding to

(A15) $$\begin{array}{c} {\hat{f}}_{s}^{eq}\left(u,1\right)∼\frac{2}{[{\langle \tau \rangle }_{+}+{\langle \tau \rangle }_{-}]\left(s+u\right)s}. \end{array}$$

Equation (A15) is easy to invert, and we find in the short time limit

(A16) $$\begin{array}{c} f_{t}^{eq}\left(T_{+},1\right)∼\frac{2}{{\langle \tau \rangle }_{+}+{\langle \tau \rangle }_{-}};\;\;\;for\;\;\;T_{+}<t. \end{array}$$

This is the short time uniformity we have found that, in turn, as explained in Section 2.2, gives the cusp like shape in P(x,t). Equation (A16) is the last term in Equation (24), corresponding to N=1 (the first two terms in Equation (24) are contributions from N=0).

Now, we are interested in inverting Equations (A10)–(A12) and then applying the definition of conditional probability Equation (A7). However, first, since we are dealing with the short time limit t⟶0, in the Laplace space, this corresponds to the limit of s⟶∞ and u⟶∞. Thus, for this particular approximation, due to the definition of the Laplace transform ${\hat{\psi }}_{\pm }\left(s\right)=\int_{0}^{\infty }e^{-st}\psi_{\pm }\left(t\right)dt$, we have that $\lim_{s\to \infty }{\hat{\psi }}_{\pm }\left(s\right)⟶0$ for a general $\psi_{\pm }\left(\tau \right)$. In this case, Equations (A10)–(A12) are approximated by

(A17) $$\begin{array}{ccc} {\hat{Q}}_{s}^{\pm }\left(1\right) & ∼ & \frac{1}{{\langle \tau \rangle }_{\pm }s^{2}}, \end{array}$$

(A18) $$\begin{array}{ccc} {\hat{f}}_{s}^{\pm }\left(u,1\right) & ∼ & \frac{1}{{\langle \tau \rangle }_{\pm }\left(s+u\right)s}. \end{array}$$

Inverting Equation (A17) with respect to *s* and Equation (A18) with respect to *u* and *s* with $0<T_{+}<t$, we obtain

(A19) $$\begin{array}{ccc} Q_{t}^{\pm }\left(1\right) & ∼ & \frac{t}{{\langle \tau \rangle }_{\pm }}, \end{array}$$

(A20) $$\begin{array}{ccc} f_{t}^{\pm }\left(T_{+},1\right) & ∼ & \frac{1}{{\langle \tau \rangle }_{\pm }}. \end{array}$$

Now, substituting Equations (A19) and (A20) in the conditional probability Equation (A7) for N=1, we obtain

(A21) $$\begin{array}{c} f_{t}^{\pm }\left(T_{+}|1\right)∼\frac{1}{t}, \end{array}$$

which is the same result shown in Equation (23) of Section 2.2. As expected, since the joint distribution $f_{t}^{\pm }\left(T_{+},N\right)$ Equation (A20) does not depend on the time or any other variable, when it is used for computing the PDF of the occupation time, it gives the uniform distribution Equation (24). The same procedure for values of N≥2 gives a joint distribution $f_{t}^{\pm }\left(T_{+},N\right)$ such that, in the double Laplace space, it is defined as

(A22) $$\begin{array}{ccc} {\hat{f}}_{s}^{+}\left(u,N\right) & = & \left(\frac{1-{\hat{\psi }}_{+}\left(s+u\right)}{{\langle \tau \rangle }_{+}\left(s+u\right)}\right){\hat{\psi }}_{-}^{k}\left(s\right){\hat{\psi }}_{+}^{k}\left(s+u\right)\left(\frac{1-{\hat{\psi }}_{-}\left(s\right)}{s}\right);\\ & if & \;\;\;N=2k+1. \end{array}$$

(A23) $$\begin{array}{ccc} {\hat{f}}_{s}^{+}\left(u,N\right) & = & \left(\frac{1-{\hat{\psi }}_{+}\left(s+u\right)}{{\langle \tau \rangle }_{+}\left(s+u\right)}\right){\hat{\psi }}_{+}^{k-1}\left(s+u\right){\hat{\psi }}_{-}^{k}\left(s\right)\left(\frac{1-{\hat{\psi }}_{+}\left(s+u\right)}{s+u}\right);\\ & if & \;\;\;N=2k. \end{array}$$

(A24) $$\begin{array}{ccc} {\hat{f}}_{s}^{-}\left(u,N\right) & = & \left(\frac{1-{\hat{\psi }}_{-}\left(s\right)}{{\langle \tau \rangle }_{-}\left(s\right)}\right){\hat{\psi }}_{-}^{k}\left(s\right){\hat{\psi }}_{+}^{k}\left(s+u\right)\left(\frac{1-{\hat{\psi }}_{+}\left(s+u\right)}{s+u}\right);\\ & if & \;\;\;N=2k+1. \end{array}$$

(A25) $$\begin{array}{ccc} {\hat{f}}_{s}^{-}\left(u,N\right) & = & \left(\frac{1-{\hat{\psi }}_{-}\left(s\right)}{{\langle \tau \rangle }_{-}\left(s\right)}\right){\hat{\psi }}_{-}^{k-1}\left(s\right){\hat{\psi }}_{+}^{k}\left(s+u\right)\left(\frac{1-{\hat{\psi }}_{-}\left(s\right)}{s}\right);\\ & if & \;\;\;N=2k. \end{array}$$

In order to analyze the joint, we have to deal with the analytical expression of $\psi_{\pm }\left(\tau \right)$ defined by Equation (12). For instance for N=2, after substituting Equation (13) in Equations (A23) and (A25), we have that the dominant term is ${\hat{f}}_{s}^{+}\left(u,2\right)∼\;\left[\Gamma \left(A_{-}+1\right)C_{A_{-}}^{-}\right]/\left[{\langle \tau \rangle }_{+}{\left(s+u\right)}^{2}s^{A_{-}+1}\right]$, and ${\hat{f}}_{s}^{-}\left(u,2\right)∼\;\left[\Gamma \left(A_{+}+1\right)C_{A_{+}}^{+}\right]/\left[{\langle \tau \rangle }_{-}s^{2}{\left(s+u\right)}^{A_{+}+1}\right]$, respectively. Inverting the double Laplace transform, in each case, it gives a positive power of $T_{+}$ and therefore, with respect to *t*, e.g., $f_{t}^{\pm }\left(T_{+},2\right)∼T_{+}^{A_{\mp }+1}$. $A_{\mp }\geq 0$ is a positive integer number, this correction term for t⟶0 (T+⟶0) is negligible, and also the remaining terms in $f_{t}^{\pm }\left(T_{+},N\right)$ with N>2. We conclude that, for the case of equilibrium initial conditions, the uniformity in the short time limit, for the PDF of the occupation/fraction time, is always preserved, as long $\psi_{\pm }\left(\tau \right)$ is analytical.

### Appendix B.1. Non-Equilibrium Initial Conditions

Still, by employing the joint distribution $f_{t}^{\pm }\left(T_{+},N\right)$, it can be shown, as follows that, for non-equilibrium initial conditions, when $\psi_{\pm }\left(\tau \right)$ in the Laplace space is approximated by ${\hat{\psi }}_{\pm }\left(s\right)∼1/s$ for short times, it can lead to a uniform distribution in the occupation time and therefore to a tent shape in P(x,t).

In the case of non-equilibrium initial conditions, either starting just from $D_{+}$ or from $D_{-}$, the averages over $\tau_{1}$, within $Q_{t}^{\pm }\left(N\right)$ Equation (A8) and the joint distribution $f_{t}^{\pm }\left(T_{+},N\right)$ Equation (A9), are no longer given by $f_{eq}^{\pm }\left(\tau_{1}\right)$ Equation (10). Now, in the non-equilibrium case, these corresponding averages are performed using the waiting time PDF $\psi_{\pm }\left(\tau_{1}\right)$. Following the same procedure as above, for the case N=1, the double Laplace transform of the joint distribution $f_{t}^{\pm }\left(T_{+},1\right)$ yields to

(A26) $$\begin{array}{c} {\hat{f}}_{s}^{\pm }\left(u,1\right)={\hat{\psi }}_{\pm }\left(s+u\right)\left(\frac{1-{\hat{\psi }}_{\mp }\left(s\right)}{s}\right). \end{array}$$

For a system with non-equilibrium initial conditions, in order to recover the uniform distribution in the PDF of $T_{+}$, it is enough to ask that, for large *s* (short times), the PDF of the waiting times in the Laplace space follows

(A27) $$\begin{array}{c} {\hat{\psi }}_{\pm }\left(s\right)∼\frac{1}{s}. \end{array}$$

Substituting Equation (A27) in Equation (A26), we have that ${\hat{f}}_{s}^{\pm }\left(u,1\right)∼1/\left[\left(s+u\right)s\right]$. This implies in the real space, for $0<T_{+}<t$, that the joint distribution follows $f_{t}^{\pm }\left(T_{+},1\right)∼1$, and therefore, because of Equation (3), we also have a uniform distribution for $T_{+}$. As an example of a model in which ${\hat{\psi }}_{\pm }\left(s\right)$ goes as Equation (A27) and for non-equilibrium initial conditions $\psi_{\pm }\left(\tau \right)\neq f_{eq}^{\pm }\left(\tau_{1}\right)$ as expected, we have the case in which the PDF of waiting times is the sum of two exponential functions, e.g., $\psi_{\pm }\left(\tau \right)=\left(1/2\right)\left\{\left[\exp\left(-\tau /C_{1\pm }\right)/C_{1\pm }\right]+\left[\exp\left(-\tau /C_{2\pm }\right)/C_{2\pm }\right]\right\}$, with $C_{1\pm },C_{2\pm }>0$. In this case for s⟶∞, ${\hat{\psi }}_{\pm }\left(s\right)∼\left[C_{1\pm }+C_{2\pm }\right]/\left[2C_{1\pm }C_{2\pm }s\right]$ and $f_{eq}^{\pm }\left(\tau_{1}\right)=\left[\exp\left(-\tau_{1}/C_{1\pm }\right)+\exp\left(-\tau_{1}/C_{2\pm }\right)\right]/\left[C_{1\pm }+C_{2\pm }\right]$. For this latter case, since ${\hat{\psi }}_{\pm }\left(s\right)$ satisfies Equation (A27), following the same analysis as above, we find that the joint distribution $f_{t}^{\pm }\left(T_{+},1\right)$ is uniform.

In Appendix D, we show that, for a system with non-equilibrium conditions and exponentially distributed waiting times with equal and different mean values, the distribution of the occupation/fraction time is also uniform. This is mainly because, for exponentially distributed waiting times, the forward recurrence time distribution in equilibrium $f_{eq}^{\pm }\left(\tau_{1}\right)$ Equation (10) is equal to $\psi_{\pm }\left(\tau_{1}\right)$, as in the non-equilibrium case. Furthermore, for exponentially distributed sojourn times, Equation (A27) is also satisfied, and its PDF in the Laplace space for s⟶∞ follows ${\hat{\psi }}_{\pm }\left(s\right)∼1/\left[{\langle \tau \rangle }_{\pm }s\right]$. By using Equation (3), this gives the uniform distribution shown in Equations (A35) and (A40).

For ${\hat{\psi }}_{\pm }\left(s\right)$ given in Equation (A27), the correction terms when N≥2, following the same analysis as in the case of equilibrium initial conditions, yield to elements of the form $f_{t}^{\pm }\left(T_{+},N\right)∼T_{+}^{N-1}$, which are negligible for $t⟶0⟺T_{+}⟶0$. Thus, they do not contribute in the PDF of the fraction occupation time.

## Appendix C. *P*(*x*,*t*) from Simulations with Uniform and Gamma Distributed Waiting Times within the Complete Range of *x*

Following Figure 3 in the left panel in which, for short time and displacements, the cusp of P(x,t) is displayed. Now, from simulations (with the same parameters as above) of a two state model, with uniform (red triangles) and gamma (blue squares) distributed waiting times. In Figure A2, we show P(x,t) in semi-log scale but for the whole span of *x*. In each case, we compare the normalized histogram of the simulation data with the short time analytical formula Equation (26), finding a perfect agreement. As we can see, the cusp is located at the origin, and, for large displacements, Gaussianity is recovered.

## Appendix D. PDF of Occupation Times for Exponentially Distributed Waiting Times and Non-Equilibrium Initial Conditions

We consider the case of a system with exponentially distributed waiting times, with ${\langle \tau \rangle }_{+}={\langle \tau \rangle }_{-}$ in Equation (31). Here, we address the situation with non-equilibrium initial conditions. Particularly, the initial conditions are such that the probability of starting at the state with $D_{+}$ is 1 and the probability of starting from the state with $D_{-}$ is 0. The PDF of $T_{+}$ then satisfies

(A28) $$\begin{array}{c} f_{t}\left(T_{+}\right)=f_{t}^{+}\left(T_{+}\right)=\sum_{N=0}^{\infty }f_{t}^{+}\left(T_{+},N\right). \end{array}$$

With $f_{t}^{+}\left(T_{+},N\right)$ given by Equation (A9) explicitly for this case, we have [46]

(A29) $$\begin{array}{ccc} f_{t}^{+}\left(T_{+},2k+1\right) & = & \int \ldots \int \delta \left(T_{+}-\sum_{i=1(odd)}^{2k+1}\tau_{i}\right)1_{\left(t_{2k+1},t_{2k+2}\right)}\left(t\right)\psi \left(\tau_{1}\right)\psi \left(\tau_{2}\right)\\ & \ldots & \psi \left(\tau_{2k+2}\right)d\tau_{1}d\tau_{2}\ldots d\tau_{2k+2}\;\;\;\;if\;\;\;\;N=2k+1,\\ f_{t}^{+}\left(T_{+},2k\right) & = & \int \ldots \int \delta \left(T_{+}-\sum_{i=1(odd)}^{2k-1}\tau_{i}-\tau^{*}\right)1_{\left(t_{2k},t_{2k+1}\right)}\left(t\right)\psi \left(\tau_{1}\right)\psi \left(\tau_{2}\right)\\ & \ldots & \psi \left(\tau_{2k+1}\right)d\tau_{1}d\tau_{2}\ldots d\tau_{2k+1}\;\;\;\;if\;\;\;\;N=2k, \end{array}$$

with $1_{\left(a,b\right)}\left(t\right)$ the indicator function equal to 1 if t∈(a,b) and 0 if t∉(a,b). We work with the double Laplace transform $L\left\{f_{t}^{+}\left(T_{+},N\right)\right\}=f_{s}^{+}\left(u,N\right)$ with t⟺s and $u⟺T_{+}$, which is given by ${\hat{f}}_{s}^{+}\left(u,N\right)=\int_{0}^{\infty }e^{-uT_{+}}\int_{0}^{\infty }e^{-st}f_{t}^{+}\left(T_{+},N\right)dtdT_{+}$. Thus, taking the double Laplace transform of Equation (A29), after substitution of ψ(τ), we have

(A30) $$\begin{array}{ccc} {\hat{f}}_{s}^{+}\left(u,2k+1\right) & = & {\hat{\psi }}^{k+1}\left(s+u\right){\hat{\psi }}^{k}\left(s\right)\left(\frac{1-\hat{\psi }\left(s\right)}{s}\right)\;\;\;\;if\;\;\;\;N=2k+1\\ {\hat{f}}_{s}^{+}\left(u,2k\right) & = & {\hat{\psi }}^{k}\left(s+u\right){\hat{\psi }}^{k}\left(s\right)\left(\frac{1-\hat{\psi }\left(s+u\right)}{s+u}\right)\;\;\;\;if\;\;\;\;N=2k. \end{array}$$

Thus, using Equation (A30) for summing over all the values of *N* in Equation (A28), we get

(A31) $$\begin{array}{ccc} {\hat{f}}_{s}\left(u\right) & = & \left(\hat{\psi }\left(s+u\right)\frac{1-\hat{\psi }\left(s\right)}{s}+\frac{1-\hat{\psi }\left(s+u\right)}{s+u}\right)\frac{1}{1-\hat{\psi }\left(s+u\right)\hat{\psi }\left(s\right)}. \end{array}$$

For exponentially distributed waiting times $\hat{\psi }\left(s\right)=1/\left(1+\langle \tau \rangle s\right)$, by substituting $\hat{\psi }\left(s\right)$ in Equation (A31), we get that the double Laplace transform of Equation (A28) is given by

(A32) $$\begin{array}{c} {\hat{f}}_{s}\left(u\right)=\frac{2+\langle \tau \rangle s}{2s+\langle \tau \rangle s^{2}+\left(1+\langle \tau \rangle s\right)u}. \end{array}$$

By the same procedures used in Appendix F, the inversion of the double Laplace transform of Equation (A32) yields

(A33) $$\begin{array}{ccc} f_{t}\left(T_{+}\right) & = & \delta \left(t-T_{+}\right)e^{-\frac{t}{\langle \tau \rangle }}+\frac{e^{-\frac{t}{\langle \tau \rangle }}}{\langle \tau \rangle }{}_{0}{\tilde{F}}_{1}\left(;1;\frac{T_{+}\left(t-T_{+}\right)}{{\langle \tau \rangle }^{2}}\right)\\ & + & \frac{e^{-\frac{t}{\langle \tau \rangle }}}{{\langle \tau \rangle }^{2}}T_{+}{}_{0}{\tilde{F}}_{1}\left(;2;\frac{T_{+}\left(t-T_{+}\right)}{{\langle \tau \rangle }^{2}}\right). \end{array}$$

Employing the identity $I_{\nu }\left(y\right)={\left(y/2\right)}^{\nu }{}_{0}{\tilde{F}}_{1}\left(;\nu +1;y^{2}/4\right)$ [61] and changing variables, we obtain the PDF of the occupation fraction, which follows

(A34) $$\begin{array}{ccc} g_{t}\left(p_{+}\right) & = & \delta \left(1-p_{+}\right)e^{-\frac{t}{\langle \tau \rangle }}+\frac{t}{\langle \tau \rangle }\{I_{0}\left(\frac{2t}{\langle \tau \rangle }\sqrt{p_{+}\left(1-p_{+}\right)}\right)\\ & + & p_{+}\frac{I_{1}\left(\frac{2t}{\langle \tau \rangle }\sqrt{p_{+}\left(1-p_{+}\right)}\right)}{\sqrt{p_{+}\left(1-p_{+}\right)}}\}e^{-\frac{t}{\langle \tau \rangle }}. \end{array}$$

By taking the series expansion of Equation (A34) in the limit t⟶0, the PDF of $p_{+}$ can be approximated by

(A35) $$\begin{array}{c} g_{t}\left(p_{+}\right)∼\delta \left(1-p_{+}\right)e^{-\frac{t}{\langle \tau \rangle }}+\frac{t}{\langle \tau \rangle }. \end{array}$$

Therefore, for $1>p_{+}>0$, the PDF of $p_{+}$ follows a uniform distribution (see the left panel of Figure A3), as in the case of equilibrium initial conditions (Equation (35)).

P(x,t) is obtained by exploiting the uniform approximation of $g_{t}\left(p_{+}\right)$ in Equation (A35), i.e.,

(A36) $$\begin{array}{c} P\left(x,t\right)∼\frac{e^{-\frac{t}{\langle \tau \rangle }-\frac{x^{2}}{4D_{+}t}}}{\sqrt{4\pi D_{+}t}}+\frac{t}{\langle \tau \rangle }\left\{\frac{2e^{-\frac{x^{2}}{4D_{+}t}}}{\sqrt{4\pi D_{+}t}}-\frac{\left|x\right|}{2D_{+}t}\left[1-Erf\left(\frac{\left|x\right|}{\sqrt{4D_{+}t}}\right)\right]\right\}. \end{array}$$

Equation (A36) follows the same structure as Equation (37), i.e., the case with equilibrium initial conditions.

When ${\langle \tau \rangle }_{+}\neq {\langle \tau \rangle }_{-}$, we have to include $\psi_{\pm }\left(\tau \right)$ and ${\hat{\psi }}_{\pm }\left(s\right)$ in Equations (A29) and (A30). Summing the resulting expressions in Equation (A28), we obtain

(A37) $$\begin{array}{ccc} {\hat{f}}_{s}\left(u\right) & = & \left({\hat{\psi }}_{+}\left(s+u\right)\frac{1-{\hat{\psi }}_{-}\left(s\right)}{s}+\frac{1-{\hat{\psi }}_{+}\left(s+u\right)}{s+u}\right)\frac{1}{1-{\hat{\psi }}_{+}\left(s+u\right){\hat{\psi }}_{-}\left(s\right)}. \end{array}$$

By employing $\hat{\psi }\left(s\right)=1/\left(1+{\langle \tau \rangle }_{\pm }s\right)$ in Equation (A37), we obtain that the double Laplace transform of the PDF of $T_{+}$ is provided by [49]

(A38) $$\begin{array}{c} {\hat{f}}_{s}\left(u\right)=\frac{{\langle \tau \rangle }_{+}+{\langle \tau \rangle }_{-}+{\langle \tau \rangle }_{+}{\langle \tau \rangle }_{-}s}{{\langle \tau \rangle }_{-}s+{\langle \tau \rangle }_{+}\left(1+{\langle \tau \rangle }_{-}s\right)\left(s+u\right)}. \end{array}$$

The inverse Laplace transform of Equation (A38) is obtained by the same procedures explained above and, in Appendix F, eventually

(A39) $$\begin{array}{ccc} g_{t}\left(p_{+}\right) & = & \delta \left(1-p_{+}\right)e^{-\frac{t}{{\langle \tau \rangle }_{+}}}+\frac{t}{{\langle \tau \rangle }_{+}}\{I_{0}\left(2t\sqrt{\frac{p_{+}\left(1-p_{+}\right)}{{\langle \tau \rangle }_{+}{\langle \tau \rangle }_{-}}}\right)\\ & + & \sqrt{\frac{{\langle \tau \rangle }_{+}}{{\langle \tau \rangle }_{-}}}p_{+}\frac{I_{1}\left(2t\sqrt{\frac{p_{+}\left(1-p_{+}\right)}{{\langle \tau \rangle }_{+}{\langle \tau \rangle }_{-}}}\right)}{\sqrt{p_{+}\left(1-p_{+}\right)}}\}e^{-\frac{tp_{+}}{{\langle \tau \rangle }_{+}}-\frac{t(1-p_{+})}{{\langle \tau \rangle }_{-}}}. \end{array}$$

We recover Equation (A34) when ${\langle \tau \rangle }_{+}={\langle \tau \rangle }_{-}=\langle \tau \rangle$. In the short time limit, Equation (A39) follows as well a uniform distribution for $1>p_{+}>0$, see the right panel of Figure A3. In this case, the PDF of $p_{+}$ is given by

(A40) $$\begin{array}{c} g_{t}\left(p_{+}\right)∼\delta \left(1-p_{+}\right)e^{-\frac{t}{{\langle \tau \rangle }_{+}}}+\frac{t}{{\langle \tau \rangle }_{+}}. \end{array}$$

Thus, for exponentially distributed waiting times, for equilibrium and non-equilibrium conditions, in the short time regime, the PDF of the occupation fraction is always uniform. This feature is only valid for exponentially distributed waiting times, and it is not necessarily fulfilled for other distributions of waiting times.

P(x,t) for the specific case of non-equilibrium initial conditions and exponentially distributed waiting times is

(A41) $$\begin{array}{c} P\left(x,t\right)∼\frac{e^{-\frac{t}{{\langle \tau \rangle }_{+}}-\frac{x^{2}}{4D_{+}t}}}{\sqrt{4\pi D_{+}t}}+\frac{t}{{\langle \tau \rangle }_{+}}\left\{\frac{e^{-\frac{x^{2}}{4D_{+}t}}}{\sqrt{\pi D_{+}t}}+\frac{\left|x\right|}{2D_{+}t}\left[1-Erf\left(\frac{\left|x\right|}{\sqrt{4D_{+}t}}\right)\right]\right\}. \end{array}$$

## Appendix E. Deduction of $g_{t}\left(p_{+}\right)$ for Waiting Times with Similar Mean Waiting Times

We can use the results found in [52,53] for inverting the double Laplace transform of $S_{t}$ as provided by Equation (33). For a Fourier–Laplace transform, $\hat{\kappa }\left(\omega ,s\right)=\int_{-\infty }^{\infty }\int_{0}^{\infty }e^{i\omega \tilde{x}}e^{-st}\kappa \left(\tilde{x},t\right)dsdx$ of the form

(A42) $$\begin{array}{c} \hat{\kappa }\left(\omega ,s\right)=\frac{2\tilde{\lambda }+s}{s^{2}+2\tilde{\lambda }s+c^{2}\omega^{2}}, \end{array}$$

the inversion yields the result [52,53]

(A43) $$\begin{array}{c} \kappa \left(\tilde{x},t\right)=\frac{1}{2}e^{-\frac{t}{\tilde{\lambda }}}\left\{\delta \left(\tilde{x}-ct\right)+\delta \left(\tilde{x}+ct\right)\right\}+\frac{1}{2\tilde{\lambda }c}\Theta (ct-|\tilde{x}\left|\right)\left[I_{0}\left(z\left(t\right)\right)+\frac{t}{\tilde{\lambda }z\left(t\right)}I_{1}\left(z\left(t\right)\right)\right], \end{array}$$

with $z\left(t\right)=\frac{1}{\tilde{\lambda }c}\sqrt{c^{2}t^{2}-{\tilde{x}}^{2}}$.

Now, we can compare the double Laplace transform of $S_{t}$ given in Equation (33) with the results shown in Equation (A42). Concretely, we can relate the Laplace variable *v* with the Fourier variable ω in $\hat{\kappa }\left(\omega ,s\right)$. Since the Laplace transform and the Fourier transform are exponential operators, by setting v=icω, we can make the former equivalent to a Fourier transform, and we use the expression given by Equation (A43) for inverting $\phi_{s}\left(v\right)$. In our case, c=1, so $S_{t}\Leftrightarrow \omega$ are Fourier conjugates and therefore the inversion of $\phi_{s}\left(v\right)$ results in

(A44) $$\begin{array}{ccc} \phi_{t}\left(S_{t}\right) & = & \frac{1}{2}e^{-\frac{t}{\langle \tau \rangle }}\{\delta \left(S_{t}-t\right)+\delta \left(S_{t}+t\right)\}\\ & + & \frac{\Theta (t-|S_{t}|)}{2\langle \tau \rangle }\left[I_{0}\left(\frac{\sqrt{t^{2}-S_{t}^{2}}}{\langle \tau \rangle }\right)+\frac{tI_{1}\left(\frac{\sqrt{t^{2}-S_{t}^{2}}}{\langle \tau \rangle }\right)}{\sqrt{t^{2}-S_{t}^{2}}}\right]. \end{array}$$

By changing variables, $S_{t}=2T_{+}-t=2p_{+}t-t$, we obtain Equation (34) in a straightforward manner. The results in Equation (34) can also be obtained by the inversion of the double Laplace transform (t⇔s and $T_{+}\Leftrightarrow u$) of the PDF of $T_{+}$ Equation (3) (see Appendix F). In this case, the double Laplace transform of the PDF of $T_{+}$ is given by

(A45) $$\begin{array}{c} {\hat{f}}_{s}\left(u\right)=\frac{4+2\langle \tau \rangle s+\langle \tau \rangle u}{2\langle \tau \rangle s^{2}+4s+\left(2+2\langle \tau \rangle s\right)u}. \end{array}$$

Finally, we mention that the moments of $T_{+}$ and therefore $p_{+}$ can be obtained by expanding Equation (A45) in powers of *u* as

(A46) $$\begin{array}{c} {\hat{f}}_{s}\left(u\right)=\frac{1}{s}-\frac{1}{2s^{2}}u+\frac{1+\langle \tau \rangle s}{2s^{3}\left(2+\langle \tau \rangle s\right)}u^{2}+O\left(u^{3}\right). \end{array}$$

The first two moments of $T_{+}$ are then

(A47) $$\begin{array}{ccc} \langle T_{+}\rangle & ∼ & \frac{t}{2}, \end{array}$$

(A48) $$\begin{array}{ccc} \langle T_{+}^{2}\rangle & ∼ & \left(\frac{\langle \tau \rangle }{4}+\frac{t}{4}\right)t+\frac{{\langle \tau \rangle }^{2}}{8}\left(e^{-\frac{2t}{\langle \tau \rangle }}-1\right). \end{array}$$

For $\langle p_{+}\rangle =\langle T_{+}\rangle /t$, we obtain

(A49) $$\begin{array}{ccc} \langle p_{+}\rangle & ∼ & \frac{1}{2}, \end{array}$$

(A50) $$\begin{array}{ccc} \langle p_{+}^{2}\rangle & ∼ & \frac{1}{4}+\frac{\langle \tau \rangle }{4t}+\frac{{\langle \tau \rangle }^{2}}{8t^{2}}\left(e^{-\frac{2t}{\langle \tau \rangle }}-1\right), \end{array}$$

(A51) $$\begin{array}{ccc} Var(p_{+}) & ∼ & \frac{\langle \tau \rangle }{4t}+\frac{{\langle \tau \rangle }^{2}}{8t^{2}}\left(e^{-\frac{2t}{\langle \tau \rangle }}-1\right). \end{array}$$

## Appendix F. Deduction of $g_{t}\left(p_{+}\right)$ for Waiting Times with ${\langle \tau \rangle }_{+}\neq {\langle \tau \rangle }_{-}$

Here, we show the procedure for obtaining Equation (45) in Section 2.3.2. Starting from the double Laplace transform of the PDF of $T_{+}$ given by Equation (44), first by inverting with respect to $u⟺T_{+}$, we get

(A52) $$\begin{array}{ccc} {\hat{f}}_{s}\left(T_{+}\right) & = & \frac{{\langle \tau \rangle }_{-}^{2}\delta \left(T_{+}\right)}{\left({\langle \tau \rangle }_{+}+{\langle \tau \rangle }_{-}\right)\left(1+{\langle \tau \rangle }_{-}s\right)}\\ & + & \frac{{\left({\langle \tau \rangle }_{+}+{\langle \tau \rangle }_{-}+{\langle \tau \rangle }_{+}{\langle \tau \rangle }_{-}s\right)}^{2}}{{\langle \tau \rangle }_{+}\left({\langle \tau \rangle }_{+}+{\langle \tau \rangle }_{-}\right){\left(1+{\langle \tau \rangle }_{-}s\right)}^{2}}e^{-T_{+}s\left(\frac{{\langle \tau \rangle }_{+}+{\langle \tau \rangle }_{-}+{\langle \tau \rangle }_{+}{\langle \tau \rangle }_{-}s}{{\langle \tau \rangle }_{+}+{\langle \tau \rangle }_{+}{\langle \tau \rangle }_{-}s}\right)}, \end{array}$$

the exponent in Equation (A52) can be written as $-T_{+}s\left(1+\frac{{\langle \tau \rangle }_{-}}{{\langle \tau \rangle }_{+}+{\langle \tau \rangle }_{+}{\langle \tau \rangle }_{-}s}\right)$. The inversion of Equation (A52) with respect to s⇔t can be expressed as

(A53) $$\begin{array}{ccc} {\hat{f}}_{s}\left(T_{+}\right) & = & \frac{{\langle \tau \rangle }_{-}e^{-\frac{t}{{\langle \tau \rangle }_{-}}}}{{\langle \tau \rangle }_{+}+{\langle \tau \rangle }_{-}}\delta \left(T_{+}\right)+L^{-1}\left\{\hat{q}\left(s\right)\hat{h}\left(s\right)\right\}. \end{array}$$

Thus, the inversion of the second term in Equation (A53) is given by the convolution theorem, following $L^{-1}\left\{\hat{q}\left(s\right)\hat{h}\left(s\right)\right\}=\int_{0}^{t}L^{-1}\left\{\hat{q}\left(s\right)\right\}{{|}_{t-t^{\prime }}L^{-1}\left\{\hat{h}\left(s\right)\right\}|}_{t^{\prime }}dt^{\prime }$, with $\hat{q}\left(s\right)=\frac{{\left({\langle \tau \rangle }_{+}+{\langle \tau \rangle }_{-}+{\langle \tau \rangle }_{+}{\langle \tau \rangle }_{-}s\right)}^{2}}{{\langle \tau \rangle }_{+}\left({\langle \tau \rangle }_{+}+{\langle \tau \rangle }_{-}\right){\left(1+{\langle \tau \rangle }_{-}s\right)}^{2}}$ and $\hat{h}\left(s\right)=e^{-T_{+}s}e^{-\frac{T_{+}{\langle \tau \rangle }_{-}s}{{\langle \tau \rangle }_{+}+{\langle \tau \rangle }_{+}{\langle \tau \rangle }_{-}s}}$. The inverse Laplace transform of $\hat{q}\left(s\right)$ is given by

(A54) $$\begin{array}{c} L^{-1}\left\{\hat{q}\left(s\right)\right\}=\frac{2e^{-\frac{t}{{\langle \tau \rangle }_{-}}}}{{\langle \tau \rangle }_{+}+{\langle \tau \rangle }_{-}}+\frac{te^{-\frac{t}{{\langle \tau \rangle }_{-}}}}{{\langle \tau \rangle }_{+}\left({\langle \tau \rangle }_{+}+{\langle \tau \rangle }_{-}\right)}+\frac{{\langle \tau \rangle }_{+}\delta \left(t\right)}{{\langle \tau \rangle }_{+}+{\langle \tau \rangle }_{-}}. \end{array}$$

The inverse Laplace transform of $\hat{h}\left(s\right)$ can be obtained by rewriting the exponent in the second term of $\hat{h}\left(s\right)$ as $-\frac{T_{+}{\langle \tau \rangle }_{-}s}{{\langle \tau \rangle }_{+}+{\langle \tau \rangle }_{+}{\langle \tau \rangle }_{-}s}=-\frac{T_{+}}{{\langle \tau \rangle }_{+}}+\frac{T_{+}{\langle \tau \rangle }_{-}}{{\langle \tau \rangle }_{+}{\langle \tau \rangle }_{-}+{\langle \tau \rangle }_{-}^{2}{\langle \tau \rangle }_{+}s}$, then we obtain

(A55) $$\begin{array}{c} L^{-1}\left\{\hat{h}\left(s\right)\right\}=L^{-1}\left\{e^{-\frac{T_{+}{\langle \tau \rangle }_{-}s}{{\langle \tau \rangle }_{+}+{\langle \tau \rangle }_{+}{\langle \tau \rangle }_{-}s}}\right\}{|}_{t-T_{+}}\Theta \left(t-T_{+}\right)\\ =e^{-\frac{T_{+}}{{\langle \tau \rangle }_{+}}}L^{-1}\left\{e^{\frac{T_{+}{\langle \tau \rangle }_{-}}{{\langle \tau \rangle }_{+}{\langle \tau \rangle }_{-}+{\langle \tau \rangle }_{-}^{2}{\langle \tau \rangle }_{+}s}}\right\}{|}_{t-T_{+}}\Theta \left(t-T_{+}\right)\\ =e^{-\frac{T_{+}}{{\langle \tau \rangle }_{+}}}[e^{-\frac{\left(t-T_{+}\right)}{{\langle \tau \rangle }_{-}}}\sqrt{\frac{T_{+}}{{\langle \tau \rangle }_{+}{\langle \tau \rangle }_{-}\left(t-T_{+}\right)}}I_{1}\left(2\sqrt{\frac{T_{+}\left(t-T_{+}\right)}{{\langle \tau \rangle }_{+}{\langle \tau \rangle }_{-}}}\right)\\ +\frac{e^{-\frac{\left(t-T_{+}\right)}{{\langle \tau \rangle }_{-}}}}{{\langle \tau \rangle }_{+}{\langle \tau \rangle }_{-}^{2}}\delta \left(\frac{t-T_{+}}{{\langle \tau \rangle }_{+}{\langle \tau \rangle }_{-}^{2}}\right)]\Theta \left(t-T_{+}\right). \end{array}$$

Substituting Equations (A54) and (A55) in Equation (A53), and, after integration, we obtain

(A56) $$\begin{array}{ccc} f_{t}\left(T_{+}\right) & = & \frac{{\langle \tau \rangle }_{-}e^{-\frac{t}{{\langle \tau \rangle }_{-}}}}{{\langle \tau \rangle }_{+}+{\langle \tau \rangle }_{-}}\delta \left(T_{+}\right)\\ & + & \frac{{\langle \tau \rangle }_{+}e^{-\frac{t}{{\langle \tau \rangle }_{+}}}}{{\langle \tau \rangle }_{+}+{\langle \tau \rangle }_{-}}\delta \left(t-T_{+}\right)+\{\frac{2}{{\langle \tau \rangle }_{+}+{\langle \tau \rangle }_{-}}{}_{0}{\tilde{F}}_{1}\left(;1;\frac{T_{+}\left(t-T_{+}\right)}{{\langle \tau \rangle }_{+}{\langle \tau \rangle }_{-}}\right)\\ & + & \left[\frac{t-T_{+}}{{\langle \tau \rangle }_{+}\left({\langle \tau \rangle }_{+}+{\langle \tau \rangle }_{-}\right)}+\frac{T_{+}}{{\langle \tau \rangle }_{-}\left({\langle \tau \rangle }_{+}+{\langle \tau \rangle }_{-}\right)}\right]{}_{0}{\tilde{F}}_{1}\left(;2;\frac{T_{+}\left(t-T_{+}\right)}{{\langle \tau \rangle }_{+}{\langle \tau \rangle }_{-}}\right)\}e^{-\frac{T_{+}}{{\langle \tau \rangle }_{+}}-\frac{\left(t-T_{+}\right)}{{\langle \tau \rangle }_{-}}}. \end{array}$$

Employing the identity $I_{\nu }\left(y\right)={\left(y/2\right)}^{\nu }{}_{0}{\tilde{F}}_{1}\left(;\nu +1;y^{2}/4\right)$ [61] and changing variables, we obtain the form of $g_{t}\left(p_{+}\right)$ as provided by Equation (45). This procedure can be employed for a system with the same mean waiting times, inverting the double Laplace transform Equation (A45) and obtaining $g_{t}\left(p_{+}\right)$ shown in Equation (34). In addition, also for the non-equilibrium cases treated in Appendix D, see Equations (A34) and (A39).

Finally, we show the corresponding first two moments of $T_{+}$ and $p_{+}$. As we proceeded in Appendix E, we obtain the moments of $T_{+}$ by expanding in powers of *u* Equation (A37), which yields

(A57) $$\begin{array}{cc} & \langle T_{+}\rangle ∼\frac{{\langle \tau \rangle }_{+}t}{{\langle \tau \rangle }_{+}+{\langle \tau \rangle }_{-}}, \end{array}$$

(A58) $$\begin{array}{cc} & \langle T_{+}^{2}\rangle ∼\frac{{\langle \tau \rangle }_{+}^{2}t^{2}}{{\left({\langle \tau \rangle }_{+}+{\langle \tau \rangle }_{-}\right)}^{2}}+\frac{2{\langle \tau \rangle }_{+}^{2}{\langle \tau \rangle }_{-}^{2}t}{{\left({\langle \tau \rangle }_{+}+{\langle \tau \rangle }_{-}\right)}^{3}}+\frac{2{\langle \tau \rangle }_{+}^{3}{\langle \tau \rangle }_{-}^{3}}{{\left({\langle \tau \rangle }_{+}+{\langle \tau \rangle }_{-}\right)}^{3}}\left(e^{-\frac{({\langle \tau \rangle }_{+}+{\langle \tau \rangle }_{-})t}{{\langle \tau \rangle }_{+}{\langle \tau \rangle }_{-}}}-1\right). \end{array}$$

Therefore, the moments of $p_{+}$ are

(A59) $$\begin{array}{cc} & \langle p_{+}\rangle ∼\frac{{\langle \tau \rangle }_{+}}{{\langle \tau \rangle }_{+}+{\langle \tau \rangle }_{-}}, \end{array}$$

(A60) $$\begin{array}{cc} & \langle p_{+}^{2}\rangle ∼\frac{{\langle \tau \rangle }_{+}^{2}}{{\left({\langle \tau \rangle }_{+}+{\langle \tau \rangle }_{-}\right)}^{2}}+\frac{2{\langle \tau \rangle }_{+}^{2}{\langle \tau \rangle }_{-}^{2}}{t{\left({\langle \tau \rangle }_{+}+{\langle \tau \rangle }_{-}\right)}^{3}}+\frac{2{\langle \tau \rangle }_{+}^{3}{\langle \tau \rangle }_{-}^{3}}{t^{2}{\left({\langle \tau \rangle }_{+}+{\langle \tau \rangle }_{-}\right)}^{3}}\left(e^{-\frac{({\langle \tau \rangle }_{+}+{\langle \tau \rangle }_{-})t}{{\langle \tau \rangle }_{+}{\langle \tau \rangle }_{-}}}-1\right), \end{array}$$

(A61) $$\begin{array}{cc} & Var\left(p_{+}\right)∼\frac{2{\langle \tau \rangle }_{+}^{2}{\langle \tau \rangle }_{-}^{2}}{t{\left({\langle \tau \rangle }_{+}+{\langle \tau \rangle }_{-}\right)}^{3}}+\frac{2{\langle \tau \rangle }_{+}^{3}{\langle \tau \rangle }_{-}^{3}}{t^{2}{\left({\langle \tau \rangle }_{+}+{\langle \tau \rangle }_{-}\right)}^{3}}\left(e^{-\frac{({\langle \tau \rangle }_{+}+{\langle \tau \rangle }_{-})t}{{\langle \tau \rangle }_{+}{\langle \tau \rangle }_{-}}}-1\right). \end{array}$$

## Appendix G. Deduction of the MSD in a Two State Model with ${\langle \tau \rangle }_{+}\neq {\langle \tau \rangle }_{-}$

From Equation (A1), we can compute the second moment of x(t) as

(A62) $$\begin{array}{ccc} \langle x^{2}\left(t\right)\rangle & = & \langle {\left(\sqrt{2D_{+}T_{+}}\xi_{1}+\sqrt{2D_{-}\left(t-T_{+}\right)}\xi_{2}\right)}^{2}\rangle ,\\ & = & 2D_{+}\langle T_{+}\rangle +2D_{-}\left(t-\langle T_{+}\rangle \right), \end{array}$$

In the second line of Equation (A62), we have employed the linearity of 〈·〉, and the properties of independent standard normal random variables, i.e., $\langle \xi_{i}^{2}\rangle =1$ and $\langle \xi_{i}\xi_{j}\rangle =0$ (with i,j∈{1,2} and i≠j). Thus, now we just have to find $\langle T_{+}\rangle$. In order to do that, we start from the definition of average occupation time

(A63) $$\begin{array}{ccc} \langle T_{+}\rangle & = & \int_{0}^{\infty }T_{+}f_{t}\left(T_{+}\right)dT_{+},\\ & = & \int_{0}^{\infty }f_{t}\left(T_{+}\right)\left(-\frac{d}{du}e^{-uT_{+}}\right){|}_{u=0}dT_{+},\\ & = & -\lim_{u\to 0}\left(\frac{d}{du}{\hat{f}}_{t}\left(u\right)\right). \end{array}$$

With ${\hat{f}}_{t}\left(u\right)=\int_{0}^{\infty }f_{t}\left(T_{+}\right)e^{-uT_{+}}$, and $T_{+}\Leftrightarrow u$ Laplace conjugates. Now, for equilibrium initial conditions, the PDF of the occupation time is given by

(A64) $$\begin{array}{c} f_{t}\left(T_{+}\right)=\frac{{\langle \tau \rangle }_{+}}{{\langle \tau \rangle }_{+}+{\langle \tau \rangle }_{-}}\sum_{N=0}^{\infty }f_{t}^{+}\left(T_{+},N\right)+\frac{{\langle \tau \rangle }_{-}}{{\langle \tau \rangle }_{+}+{\langle \tau \rangle }_{-}}\sum_{N=0}^{\infty }f_{t}^{-}\left(T_{+},N\right), \end{array}$$

with $f_{t}^{\pm }\left(T_{+},N\right)$ the joint PDF of the occupation times at $D_{+}$ and the number of jumps between states during *t*, once started from $D_{\pm }$. When starting from $D_{+}$ and having N=2k+1 or N=2k jumps, the occupation time in each case satisfies Equation (1). In the case when the initial state is at $D_{-}$, we have

(A65) $$\begin{array}{ccc} T_{+} & = & \tau_{2}+\tau_{4}+\ldots +\tau^{*}\;\;\;\;\;\;\;\;\;\;\;\;if\;\;\;\;N=2k+1,\\ T_{+} & = & \tau_{2}+\tau_{4}+\ldots +\tau_{N}\;\;\;\;\;\;\;\;\;\;\;if\;\;\;\;N=2k, \end{array}$$

with $\tau^{*}=t-t_{N}$, the backward recurrence time. The definition of the joint PDF $f_{t}^{\pm }\left(T_{+},N\right)$ is already given in Equation (A9). In addition, its double Laplace transform ${\hat{f}}_{s}^{\pm }\left(u,N\right)=\int_{0}^{\infty }\int_{0}^{\infty }f_{t}\left(T_{+},N\right)\exp\left(-uT_{+}-st\right)\;dT_{+}\;dt$, is shown in Equations (A22)–(A25). When N=0, we have

(A66) $$\begin{array}{ccc} {\hat{f}}_{s}^{+}\left(u,0\right) & = & \frac{1-\left(\frac{1-{\hat{\psi }}_{+}\left(s+u\right)}{{\langle \tau \rangle }_{+}\left(s+u\right)}\right)}{s+u},\\ {\hat{f}}_{s}^{-}\left(u,0\right) & = & \frac{1-\left(\frac{1-{\hat{\psi }}_{-}\left(s\right)}{{\langle \tau \rangle }_{-}s}\right)}{s}. \end{array}$$

Now, for obtaining ${\hat{f}}_{s}\left(u\right)$, we compute the double Laplace transform of Equation (A64) and then we sum Equations (A22), (A25), and (A66) for all values of *N*. Thereafter, we compute the derivative of ${\hat{f}}_{s}\left(u\right)$ with respect to *u* and its corresponding limit when u⟶0. Following algebraic simplifications, we yield

(A67) $$\begin{array}{c} \lim_{u\to 0}\left(\frac{d}{du}{\hat{f}}_{s}\left(u\right)\right)=-\frac{{\langle \tau \rangle }_{+}}{{\langle \tau \rangle }_{+}+{\langle \tau \rangle }_{-}}\frac{1}{s^{2}}. \end{array}$$

For obtaining the average occupation time Equation (A63), we invert Equation (A67) with respect to *s*, having

(A68) $$\begin{array}{c} \langle T_{+}\rangle =\left(\frac{{\langle \tau \rangle }_{+}}{{\langle \tau \rangle }_{+}+{\langle \tau \rangle }_{-}}\right)t. \end{array}$$

Finally, substituting Equation (A68) in Equation (A62), we get Equation (49), which indicates that the MSD is linear with respect to *t*, for any value of time *t*.

## References

[1] Chaudhuri P., Berthier L., Kob W. (2007). Universal Nature of Particle Displacements close to Glass and Jamming Transitions. Phys. Rev. Lett..

[2] Hapca S., Crawford J.W., Young I.M. (2009). Anomalous diffusion of heterogeneous populations characterized by normal diffusion at the individual level. J. R. Soc. Interface.

[3] Wang B., Anthony S.M., Bae S.C., Granick S. (2009). Anomalous yet Brownian. Proc. Natl. Acad. Sci..

[4] Leptos K.C., Guasto J.S., Gollub J.P., Pesci A.I., Goldstein R.E. (2009). Dynamics of Enhanced Tracer Diffusion in Suspensions of Swimming Eukaryotic Microorganisms. Phys. Rev. Lett..

[5] Wang B., Kuo J., Bae S.C., Granick S. (2012). When Brownian diffusion is not Gaussian. Nat. Mater..

[6] Lampo T.J., Stylianidou S., Backlund M.P., Wiggins P.A., Spakowitz A.J. (2017). Cytoplasmic RNA-Protein Particles Exhibit Non-Gaussian Subdiffusive Behavior. Biophys J..

[7] Sabri A., Xu X., Krapf D., Weiss M. (2020). Elucidating the Origin of Heterogeneous Anomalous Diffusion in the Cytoplasm of Mammalian Cells. Phys. Rev. Lett..

[8] Weeks E.R., Crocker J.C., Levitt A.C., Schofield A., Weitz D.A. (2000). Three-Dimensional Direct Imaging of Structural Relaxation Near the Colloidal Glass Transition. Science.

[9] Kegel W.K., van Blaaderen A. (2000). Direct Observation of Dynamical Heterogeneities in Colloidal Hard-Sphere Suspensions. Science.

[10] Chakraborty I., Roichman Y. (2020). Disorder-induced Fickian, yet non-Gaussian diffusion in heterogeneous media. Phys. Rev. Res..

[11] Lavaud M., Salez T., Louyer Y., Amarouchene Y. (2020). Surface Force Measurements Using Brownian Particles. arXiv.

[12] Chubynsky M.V., Slater G.W. (2014). Diffusing Diffusivity: A Model for Anomalous, yet Brownian, Diffusion. Phys. Rev. Lett..

[13] Miyaguchi T., Akimoto T., Yamamoto E. (2016). Langevin equation with fluctuating diffusivity: A two-state model. Phys. Rev. E.

[14] Chechkin A.V., Seno F., Metzler R., Sokolov I.M. (2017). Brownian yet Non-Gaussian Diffusion: From Superstatistics to Subordination of Diffusing Diffusivities. Phys. Rev. X.

[15] Sposini V., Chechkin A.V., Seno F., Pagnini G., Metzler R. (2018). Random diffusivity from stochastic equations: Comparison of two models for Brownian yet non-Gaussian diffusion. New J. Phys..

[16] Lanoiselée Y., Grebenkov D.S. (2018). A model of non-Gaussian diffusion in heterogeneous media. J. Phys. A.

[17] Sposini V., Chechkin A., Metzler R. (2018). First, passage statistics for diffusing diffusivity. J. Phys. A.

[18] Ślęzak J., Burnecki K., Metzler R. (2019). Random coefficient autoregressive processes describe Brownian yet non-Gaussian diffusion in heterogeneous systems. New J. Phys..

[19] Grebenkov D., Sposini V., Metzler R., Oshanin G., Seno F. (2020). Exact first-passage time distributions for three random diffusivity models. J. Phys. A.

[20] Wang W., Seno F., Sokolov I.M., Chechkin A.V., Metzler R. (2020). Unexpected crossovers in correlated random-diffusivity processes. New J. Phys..

[21] Jain R., Sebastian K.L. (2016). Diffusion in a Crowded, Rearranging Environment. J. Phys. Chem. B.

[22] Barkai E., Burov S. (2020). Packets of Diffusing Particles Exhibit Universal Exponential Tails. Phys. Rev. Lett..

[23] Wang W., Barkai E., Burov S. (2020). Large Deviations for Continuous Time Random Walks. Entropy.

[24] Pacheco-Pozo A., Sokolov I.M. (2020). Large Deviation in Continuous Time Random Walks. arXiv.

[25] Samanta N., Chakrabarti R. (2016). Tracer diffusion in a sea of polymers with binding zones: Mobile vs. frozen traps. Soft Matter.

[26] Kumar P., Theeyancheri L., Chaki S., Chakrabarti R. (2019). Transport of probe particles in a polymer network: Effects of probe size, network rigidity and probe–polymer interaction. Soft Matter.

[27] Baldovin F., Orlandini E., Seno F. (2019). Polymerization Induces Non-Gaussian Diffusion. Front. Phys..

[28] Hidalgo-Soria M., Barkai E. (2020). Hitchhiker model for Laplace diffusion processes. Phys. Rev. E.

[29] Yin Q., Li Y., Marchesoni F., Nayak S., Ghosh P. (2021). Non-Gaussian Normal Diffusion in Low Dimensional Systems. arXiv.

[30] Goswami K., Sebastian K.L. (2020). Exact solution to the first-passage problem for a particle with a dichotomous diffusion coefficient. Phys. Rev. E.

[31] Bouchaud J., Comtet A., Georges A., Le Doussal P. (1990). Classical diffusion of a particle in a one-dimensional random force field. Ann. Phys..

[32] Monthus C. (2003). Anomalous diffusion, localization, aging, and subaging effects in trap models at very low temperature. Phys. Rev. E.

[33] Burov S., Barkai E. (2011). Time Transformation for Random Walks in the Quenched Trap Model. Phys. Rev. Lett..

[34] Burov S., Barkai E. (2012). Weak subordination breaking for the quenched trap model. Phys. Rev. E.

[35] Luo L., Yi M. (2018). Non-Gaussian diffusion in static disordered media. Phys. Rev. E.

[36] Luo L., Yi M. (2019). Quenched trap model on the extreme landscape: The rise of subdiffusion and non-Gaussian diffusion. Phys. Rev. E.

[37] Postnikov E.B., Chechkin A., Sokolov I.M. (2020). Brownian yet non-Gaussian diffusion in heterogeneous media: From superstatistics to homogenization. New J. Phys..

[38] Regev S., Grønbech-Jensen N., Farago O. (2016). Isothermal Langevin dynamics in systems with power-law spatially dependent friction. Phys. Rev. E.

[39] Radice M., Onofri M., Artuso R., Cristadoro G. (2019). Transport properties and ageing for the averaged Lévy–Lorentz gas. J. Phys..

[40] Barkai E. (2001). Fractional Fokker–Planck equation, solution, and application. Phys. Rev. E.

[41] Kärger J. (1985). NMR self-diffusion studies in heterogeneous systems. Adv. Colloid Interface Sci..

[42] Margolin G., Barkai E. (2004). Aging correlation functions for blinking nanocrystals, and other on–off stochastic processes. J. Chem. Phys..

[43] Aharony A., Entin-Wohlman O., Chowdhury D., Dattagupta S. (2019). Is Telegraph Noise A Good Model for the Environment of Mesoscopic Systems?. J. Stat. Phys..

[44] Yamamoto E., Akimoto T., Mitsutake A., Metzler R. (2020). Universal relation between instantaneous diffusivity and radius of gyration of proteins in aqueous solution. arXiv.

[45] Kanazawa K., Sano T.G., Cairoli A., Baule A. (2020). Loopy Lévy flights enhance tracer diffusion in active suspensions. Nature.

[46] Godrèche C., Luck J.M. (2001). Statistics of the Occupation Time of Renewal Processes. J. Stat. Phys..

[47] Miyaguchi T., Uneyama T., Akimoto T. (2019). Brownian motion with alternately fluctuating diffusivity: Stretched-exponential and power-law relaxation. Phys. Rev. E.

[48] Cox D.R. (1962). Renewal Theory.

[49] Bel G., Barkai E. (2005). Occupation times and ergodicity breaking in biased continuous time random walks. J. Phys. Condens. Matter.

[50] Schulz J.H.P., Barkai E., Metzler R. (2013). Aging Effects and Population Splitting in Single-Particle Trajectory Averages. Phys. Rev. Lett..

[51] Schulz J.H.P., Barkai E., Metzler R. (2014). Aging Renewal Theory and Application to Random Walks. Phys. Rev. X.

[52] Masoliver J., Weiss G.H. (1996). Finite-velocity diffusion. Eur. J. Phys..

[53] Masoliver J., Lindenberg K. (2017). Continuous time persistent random walk: A review and some generalizations. Eur. Phys. J. B.

[54] Beck C., Cohen E. (2003). Superstatistics. Phys. A Stat. Mech. Appl..

[55] Laplace P.S. (1774). Mémoirs présentés à l’ Académie des Sciences.

[56] Wilson E.B. (1923). First, and Second Laws of Error. J. Am. Stat. Assoc..

[57] Burov S., Jeon J.H., Metzler R., Barkai E. (2011). Single particle tracking in systems showing anomalous diffusion: The role of weak ergodicity breaking. Phys. Chem. Chem. Phys..

[58] Metzler R., Jeon J.H., Cherstvy A.G., Barkai E. (2014). Anomalous diffusion models and their properties: Non-stationarity, non-ergodicity, and ageing at the centenary of single particle tracking. Phys. Chem. Chem. Phys..

[59] Grebenkov D.S. (2019). Time-averaged mean square displacement for switching diffusion. Phys. Rev. E.

[60] Wang W., Cherstvy A.G., Chechkin A.V., Thapa S., Seno F., Liu X., Metzler R. (2020). Fractional Brownian motion with random diffusivity: Emerging residual nonergodicity below the correlation time. J. Phys. A.

[61] From Wolfram Research. https://functions.wolfram.com/Bessel-TypeFunctions/BesselI/26/01/01/.

